# Crucial Role of Mesangial Cell-derived Connective Tissue Growth Factor in a Mouse Model of Anti-Glomerular Basement Membrane Glomerulonephritis

**DOI:** 10.1038/srep42114

**Published:** 2017-02-13

**Authors:** Naohiro Toda, Kiyoshi Mori, Masato Kasahara, Akira Ishii, Kenichi Koga, Shoko Ohno, Keita P. Mori, Yukiko Kato, Keisuke Osaki, Takashige Kuwabara, Katsutoshi Kojima, Daisuke Taura, Masakatsu Sone, Taiji Matsusaka, Kazuwa Nakao, Masashi Mukoyama, Motoko Yanagita, Hideki Yokoi

**Affiliations:** 1 Department of Nephrology, Graduate School of Medicine, Kyoto University, Kyoto Japan; 2 School of Pharmaceutical Sciences, University of Shizuoka, Shizuoka, Japan; 3 Department of Nephrology and Kidney Research, Shizuoka General Hospital, Shizuoka, Japan; 4 Institute for Clinical and Translational Science, Nara Medical University Hospital, Kashihara, Japan; 5 Department of Nephrology, Kumamoto University Graduate School of Medical Sciences, Kumamoto, Japan; 6 Department of Diabetes, Endocrinology and Nutrition, Graduate School of Medicine, Kyoto University, Kyoto, Japa; 7 Department of Molecular Life Sciences, Tokai University School of Medicine, Isehara, Japan; 8 Medical Innovation Center, Graduate School of Medicine, Kyoto University, Kyoto Japan

## Abstract

Connective tissue growth factor (CTGF) coordinates the signaling of growth factors and promotes fibrosis. Neonatal death of systemic CTGF knockout (KO) mice has hampered analysis of CTGF in adult renal diseases. We established 3 types of CTGF conditional KO (cKO) mice to investigate a role and source of CTGF in anti-glomerular basement membrane (GBM) glomerulonephritis. Tamoxifen-inducible systemic CTGF (Rosa-CTGF) cKO mice exhibited reduced proteinuria with ameliorated crescent formation and mesangial expansion in anti-GBM nephritis after induction. Although CTGF is expressed by podocytes at basal levels, podocyte-specific CTGF (pod-CTGF) cKO mice showed no improvement in renal injury. In contrast, PDGFRα promoter-driven CTGF (*Pdgfra*-CTGF) cKO mice, which predominantly lack CTGF expression by mesangial cells, exhibited reduced proteinuria with ameliorated histological changes. Glomerular macrophage accumulation, expression of *Adgre1* and *Ccl2*, and ratio of M1/M2 macrophages were all reduced both in Rosa-CTGF cKO and *Pdgfra*-CTGF cKO mice, but not in pod-CTGF cKO mice. TGF-β1-stimulated *Ccl2* upregulation in mesangial cells and macrophage adhesion to activated mesangial cells were decreased by reduction of CTGF. These results reveal a novel mechanism of macrophage migration into glomeruli with nephritis mediated by CTGF derived from mesangial cells, implicating the therapeutic potential of CTGF inhibition in glomerulonephritis.

Anti-glomerular basement membrane glomerulonephritis (anti-GBM nephritis) is a life-threatening disease^1^,^2^. Monocyte chemoattractant protein-1 (MCP-1 or CCL2) and transforming growth factor-β (TGF-β) are reported to be major mediators of progressive anti-GBM nephritis^3^,^4^,^5^. Connective tissue growth factor (CTGF, also known as CCN2) is a 36–38 kDa protein and has been shown to be a downstream mediator of the profibrotic property of TGF-β^6^,^8^. CTGF exerts multiple physiological actions, including extracellular matrix (ECM) accumulation, cell proliferation, cell adhesion and migration^7^,^8^,^9^,^10^,^11^,^12^.

In the kidney, CTGF mRNA is weakly expressed by podocytes and parietal epithelial cells under normal conditions^13^. CTGF is upregulated in mesangial proliferative lesions and crescents in patients with crescentic glomerulonephritis^13^. Previously, we demonstrated that podocyte-specific CTGF overexpression in mice leads to glomerular injury in a streptozotocin–induced model of diabetes^14^, and that knockdown of CTGF gene expression ameliorates tubulointerstitial fibrosis in obstructive nephropathy^15^, indicating that CTGF is a mediator of renal fibrosis *in vivo*.

However, identification of the specific glomerular cell types responsible for pathological CTGF expression has remained unanswered. In the present study, we generated tamoxifen-inducible CTGF conditional KO (Rosa-CTGF cKO) mice to investigate importance of CTGF in the progression of anti-GBM nephritis, since conventional systemic CTGF KO mice die shortly after birth because of respiratory failure caused by skeletal defects^16^. Furthermore, we generated podocyte-specific CTGF conditional KO (pod-CTGF cKO) mice. Thirdly, we also established PDGFRα promoter-driven CTGF conditional KO (*Pdgfra*-CTGF cKO) mice, which predominantly lack CTGF expression in mesangial cells of the glomeruli, to investigate the cell type-specific contribution of CTGF in anti-GBM nephritis model.

## Results

### Generation of CTGF floxed mice

To inactivate the *Ctgf* gene, we generated mice harboring *Ctgf*^*flox*^ (*Ctgf*^*fl*^) allele, which allows deletion of the entire *Ctgf* coding sequences (Supplementary Fig. S1a). We crossed *Ctgf*^*fl/fl*^ mice with ROSA26-CreER^T2^ mice to generate tamoxifen-inducible systemic *Ctgf* conditional KO (Rosa-CTGF cKO) mice. Southern blot analysis showed efficient deletion of *Ctgf* gene in the kidney of male ROSA26-CreER^T2^; *Ctgf*^*fl/fl*^ mice treated with 4-hydroxytamoxifen (4-OHT, 0.05 mg/kgBW, three times) when they were 3 weeks of age (Supplementary Fig. S1b). Gene expression of *Ctgf* in the kidneys of Rosa-CTGF cKO mice was decreased by 80% (Supplementary Fig. S1c) and *Ctgf* expression was also reduced in the heart, liver, and lungs (Supplementary Fig. S1d). Rosa-CTGF cKO mice exhibited healthy gross appearance with normal histology of the kidney, heart, liver, and lung (Supplementary Fig. S1e).

### Rosa-CTGF cKO mice exhibited reduced proteinuria in anti-GBM nephritis model

**Figure 1:**
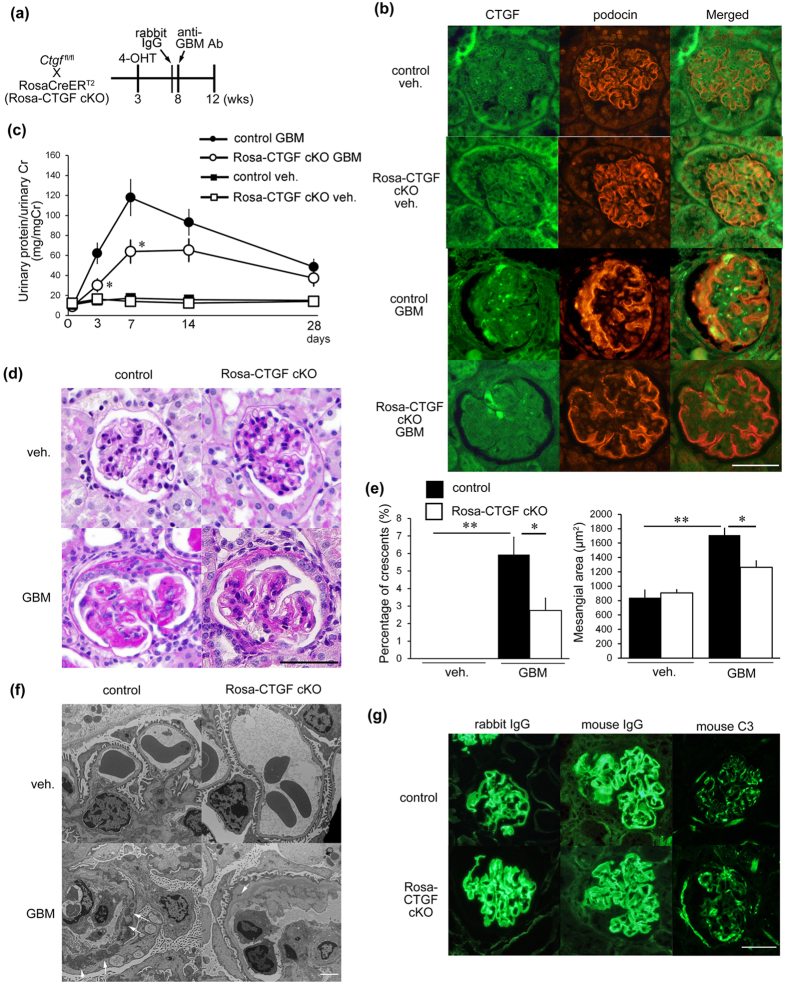
Deletion of CTGF ameliorated proteinuria and histological changes in anti-GBM nephritis. (**a**) An experimental protocol for the study on the anti-GBM nephritis in tamoxifen-inducible systemic CTGF cKO (Rosa-CTGF cKO) mice. Three-week old male ROSA26-CreER^T2^; *Ctgf*^*fl/fl*^ mice or control [Cre (−); *Ctgf*^*fl/fl*^] were intraperitoneally injected with 4-OHT, and anti-GBM nephritis was induced at 8 weeks of age (GBM). Vehicle treatment was also carried out in some animals (veh.). Mice were killed at 4 weeks after induction of anti-GBM nephritis. (**b**) Double immunofluorescence staining for CTGF (green) and podocin (red) in the glomeruli of control or Rosa-CTGF cKO mice at 4 weeks after induction of anti-GBM nephritis. Bar represents 50 μm. (**c**) Changes in proteinuria during the course of anti-GBM nephritis. Closed squares, vehicle-treated control mice; open square, vehicle-treated Rosa-CTGF cKO mice; closed circles, control mice with GBM nephritis; open circles, Rosa-CTGF cKO mice with anti-GBM nephritis. (**d**) Representative photomicrographs of the kidneys at 4 weeks after induction of anti-GBM nephritis (PAS staining). Left upper panel, vehicle-treated control mice; right upper panel, vehicle-treated Rosa-CTGF cKO mice; left lower panel, control mice with anti-GBM nephritis; right lower panel show, Rosa-CTGF cKO mice with anti-GBM nephritis. Bar represents 50 μm. (**e**) The percentage of crescent formation (left panel) and the mesangial area (right panel) in anti-GBM nephritis. (**f**) Electron microscopic analysis of glomeruli. Bar represents 2,000 nm. (**g**) Immunofluorescence studies for rabbit IgG, mouse IgG and mouse C3 deposition in the kidney at 28 days after induction of anti-GBM nephritis in control and in Rosa-CTGF cKO mice. Both Rosa-CTGF cKO and control mice exhibited liner IgG and C3 deposition along the GBM. Bar represents 50 μm. Values are expressed as means ± s.e. ^*^P < 0.05, ^**^P < 0.01 vs control mice with GBM nephritis. Vehicle-treated control mice (n = 7), vehicle-treated Rosa-CTGF cKO mice (n = 7), control mice with GBM nephritis (n = 7), Rosa-CTGF mice with GBM nephritis (n = 9). Veh, vehicle; GBM, anti-GBM nephritis.

To examine a role of CTGF in glomerulonephritis, we induced anti-GBM nephritis in Rosa-CTGF cKO and control mice. At 8 weeks of age, 4-OHT-pretreated mutant and control mice were administered with anti-GBM serum (Fig. 1a), and renal examination was conducted 4 weeks later. CTGF was expressed weakly by podocytes and mesangial cells in normal glomeruli (Fig. 1b, Supplementary Fig. S2), and its expression was almost same as Rosa-CTGF cKO mice without nephritis (vehicle). Induction of anti-GBM nephritis resulted in increase of CTGF protein expression predominantly in the glomeruli, with a degree of co-localization with podocin, a podocyte marker (Fig. 1b, Supplementary Fig. S2). On the other hand, Rosa-CTGF cKO mice with anti-GBM nephritis exhibited a decrease in CTGF expression by podocytes and mesangial cells (Fig. 1b, Supplementary Fig. S2). No difference in urinary protein was observed between the vehicle-treated control and Rosa-CTGF cKO mice (Fig. 1c). Control mice with anti-GBM nephritis developed massive proteinuria that peaked at 1 week and decreased gradually thereafter (Fig. 1c). In contrast, Rosa-CTGF cKO mice with anti-GBM nephritis exhibited significantly reduced proteinuria at 1 week (Fig. 1c).

Microscopic examination indicated increased crescent formation and mesangial expansion in control mice with glomerulonephritis at 4 weeks (Fig. 1d). These changes were significantly ameliorated in Rosa-CTGF cKO mice with nephritis (Fig. 1d). Both control and Rosa-CTGF cKO mice with nephritis showed slight fibrotic changes in interstitial areas (Supplementary Fig. S3). Electron microscopic analysis demonstrated that Rosa-CTGF cKO mice without nephritis exhibited almost normal glomerular structure (Fig. 1f). Control mice with nephritis exhibited dense deposit accumulation along the GBM, and deposition was significantly reduced in Rosa-CTGF cKO mice with nephritis (Fig. 1f). Both Rosa-CTGF cKO mice and control mice showed liner rabbit and mouse IgG and C3 deposition along the GBM (Fig. 1g). These findings indicate that the deletion of CTGF did not affect the process of heterologous antibody-complement deposition or autologous antibody production.

**Figure 2:**
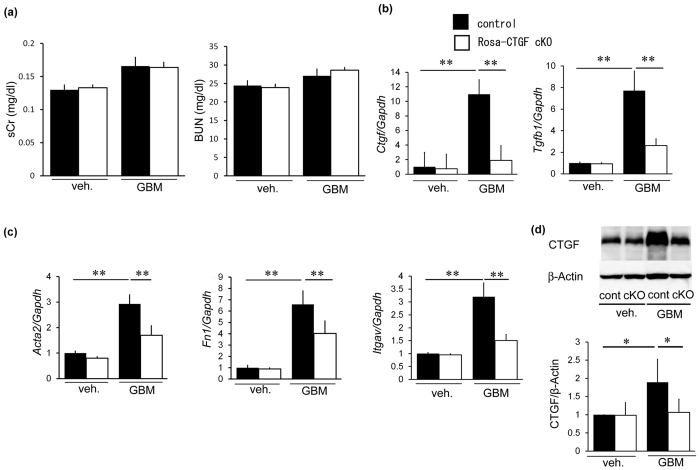
Systemic deletion of CTGF reduced mRNA expression of fibrotic makers in anti-GBM nephritis. (**a**) Serum creatinine and BUN levels. No significant difference between control and Rosa-CTGF cKO mice with nephritis. (**b**) Expression of *Ctgf* and *Tgfb1* mRNA in the glomeruli of the kidney at 4 weeks after induction of anti-GBM nephritis. Glomerular *Ctgf* expression decreased by 80% in Rosa-CTGF cKO mice with nephritis compared with control mice with nephritis. *Tgfb1* expression was also decreased in Rosa-CTGF cKO with nephritis. (**c**) Expression of *Acta2, Fn1 and Itgav* mRNA in the glomeruli. *Gapdh* mRNA expression was used as internal control. (**d**) Glomerular CTGF protein at 4 weeks after induction of anti-GBM nephritis by Western blotting (n = 4, each). β-actin was used as internal control. Full-length blots are presented in Supplementary Figure S10. Values are expressed as means ± s.e. ^*^P < 0.05, ^**^P < 0.01. Vehicle-treated control mice (n = 7), vehicle-treated Rosa-CTGF cKO mice (n = 7), control mice with anti-GBM nephritis (n = 7), Rosa-CTGF cKO mice with anti-GBM nephritis (n = 9). Veh, vehicle; GBM, anti-GBM nephritis.

Serum creatinine and blood urea nitrogen (BUN) levels were not different between control and Rosa-CTGF cKO mice with nephritis (Fig. 2a). Glomerular *Ctgf* mRNA expression in control mice with anti-GBM nephritis was 11 times higher than in control mice without nephritis (Fig. 2b). Glomerular *Ctgf* expression decreased by 80% in Rosa-CTGF cKO mice with nephritis. Glomerular gene expression of *Tgfb1*, α-smooth muscle actin (*Acta2)*, fibronectin (*Fn1)* and integrin αv (*Itgav*) was increased in control mice with nephritis and this increase was reduced in Rosa-CTGF cKO mice with nephritis (Fig. 2b,c). Glomerular CTGF protein of Rosa-CTGF cKO mice in GBM nephritis was also reduced compared with that of control mice (Fig. 2d). These results demonstrate that systemic deletion of CTGF ameliorates proteinuria and glomerular injury in anti-GBM nephritis.

### Podocyte-specific inhibition of CTGF did not affect glomerulonephritis

**Figure 3:**
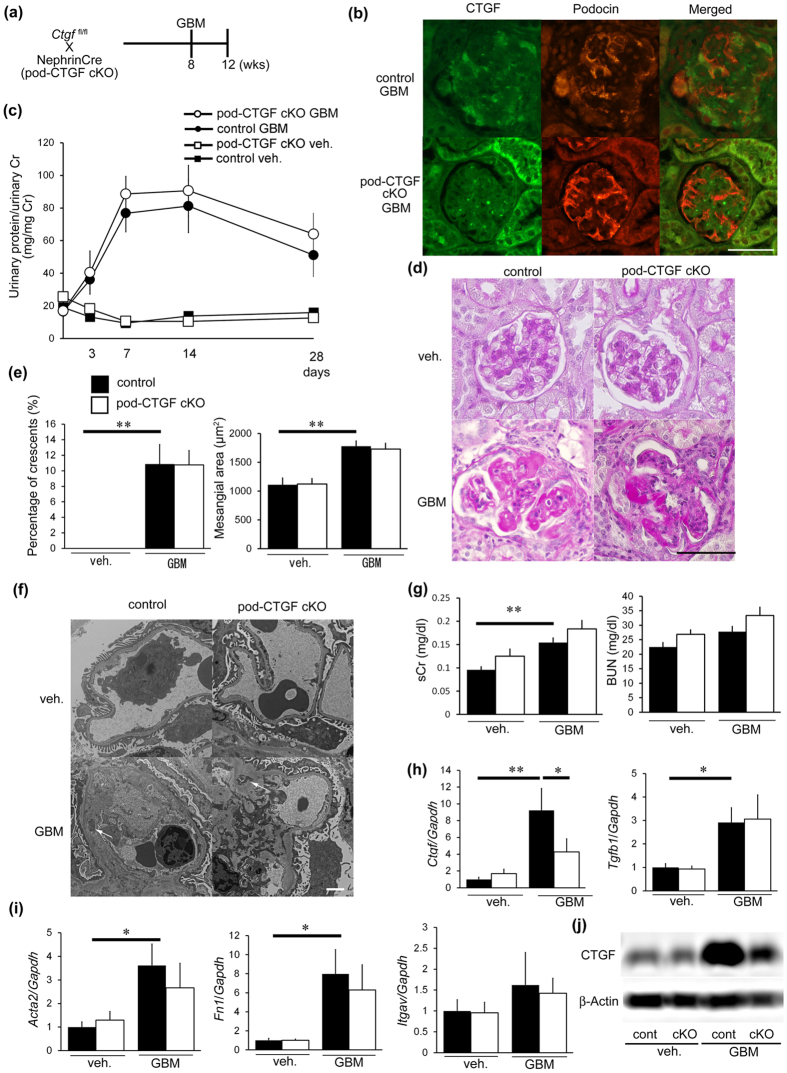
Podocyte-specific inhibition of CTGF did not ameliorate glomerular changes. (**a**) Experimental protocol for anti-GBM nephritis in podocyte-specific CTGF cKO mice (pod-CTGF cKO). (**b**) Double immunofluorescence staining for CTGF (green) and podocin (red) in glomeruli of pod-CTGF cKO mice at 4 weeks after induction of anti-GBM nephritis. Pod-CTGF cKO mice with anti-GBM nephritis exhibited a decrease in CTGF expression by podocytes. Lower panel, pod-CTGF cKO mice with anti-GBM nephritis. Bar represents 50 μm. (**c**) Changes in proteinuria during the course of the anti-GBM nephritis. Closed squares, vehicle-treated control mice; open squares, pod-CTGF cKO mice with vehicle; closed circles, control mice with anti-GBM nephritis; open circles, pod-CTGF cKO mice with anti-GBM nephritis. (**d**) Representative photomicrographs of the kidneys at 4 weeks after induction of anti-GBM nephritis (PAS staining). Left upper panel, vehicle-treated control mice; right upper panel, vehicle-treated pod-CTGF cKO mice; left lower panel, control mice with anti-GBM nephritis; right lower panel, pod-CTGF cKO mice with anti-GBM nephritis. Bar represents 50 μm. (**e**) The percentage of crescent formation (left panel) and the mesangial area (right panel) in anti-GBM nephritis in pod-CTGF cKO mice. (**f**) Electron microscopic analysis of glomeruli in anti-GBM nephritis in pod-CTGF cKO mice. No difference in dense deposit accumulation (white arrows) between control and pod-CTGF cKO mice with nephritis. Bar represents 2,000 nm. (**g**) Serum creatinine and BUN levels in pod-CTGF cKO mice with anti-GBM nephritis. (**h**) Expression of *Ctgf* and *Tgfb1* mRNA in glomeruli at 4 weeks after induction of anti-GBM nephritis. (**i**) Expression of *Acta2, Fn1*and *Itgav* mRNA in glomeruli. (**j**) Representative image of glomerular CTGF protein by Western blotting. Immunoblot of β-actin was used as control. Full-lengh blots are presented in Supplementary Figure S11. Values are expressed as means ± s.e. ^*^P < 0.05, ^**^P < 0.01. Vehicle-treated control mice (n = 9), vehicle-treated pod-CTGF cKO mice (n = 6), control mice with anti-GBM nephritis (n = 7), pod-CTGF mice with anti-GBM nephritis (n = 7). Veh, vehicle; GBM, anti-GBM nephritis.

To examine the contribution of glomerular cell types as sources of CTGF, we focused on podocytes because CTGF is expressed by podocytes at basal levels. We generated podocyte-specific CTGF cKO (pod-CTGF cKO) mice and induced anti-GBM nephritis (Fig. 3a). Pod-CTGF cKO mice with anti-GBM nephritis exhibited a decrease in CTGF expression by podocytes (Fig. 3b). No difference in urinary protein excretion was observed between vehicle-treated control and pod-CTGF cKO mice (Fig. 3c). In glomerulonephritis, pod-CTGF cKO mice did not show improvements in proteinuria (Fig. 3c). Histological changes with nephritis, including crescent formation and mesangial expansion, in pod-CTGF cKO mice were not significantly different from control mice with nephritis (Fig. 3d,e). Electron microscopic analysis showed normal glomerular structure, including podocyte morphology, in pod-CTGF cKO mice without nephritis (Fig. 3f). Both control and pod-CTGF cKO mice with nephritis showed similar dense deposit accumulation along the GBM (Fig. 3f), with no significant difference of serum creatinine and BUN levels (Fig. 3g).

In control mice with nephritis, *Ctgf, Tgfb1, Acta2, Fn1,* and *Itgav* mRNA expressions in the glomeruli were increased compared with control mice without nephritis (Fig. 3h,i). Glomerular *Ctgf* expression decreased by 50% in pod-CTGF cKO mice with nephritis compared with control mice with nephritis. No significant difference in the expression of other mRNA transcripts was observed between control and pod-CTGF cKO mice with nephritis (Fig. 3h,i). Glomerular CTGF protein was also reduced in pod-CTGF cKO mice with nephritis (Fig. 3j). These results indicate that podocyte-specific inhibition of CTGF does not ameliorate proteinuria and glomerular injury in anti-GBM nephritis.

### Reduction of CTGF in mesangial cells ameliorated glomerulonephritis

Next, we focused on mesangial cells and generated *Pdgfra*-CTGF cKO mice to eliminate CTGF in mesangial cells. PDGFRα was predominantly expressed by mesangial cells in normal glomeruli, and its expression was increased after induction of anti-GBM nephritis (Supplementary Fig. S4a). *Pdgfra*-ZsGreen mice showed that ZsGreen was localized in cells positive for PDGFRβ, a representative mesangial and fibroblast marker, and was not co-localized with podocin or an endothelial marker PECAM1 (Supplementary Fig. S4b,c), and that ZsGreen was partly co-localized with cells positive for Megalin (Supplementary Fig. S4d), a proximal tubular cell marker, and aquaporin 2 (AQP2), a collecting duct cell marker (Supplementary Fig. S4e). These results indicate that PDGFRα was predominantly expressed by mesangial cells within glomeruli in anti-GBM nephritis. *Pdgfra*-ZsGreen mice in control exhibited a few ZsGreen-positive cells in bone marrow (Supplementary Fig. S5a). These ZsGreen-positive cells sorted by flow cytometry expressed little *Ctgf* mRNA (Supplementary Fig. S5b), suggesting that *Pdgfra*-CTGF cKO mice do not have any changes in *Ctgf* mRNA expression in bone marrow cells compared with control mice.

**Figure 4:**
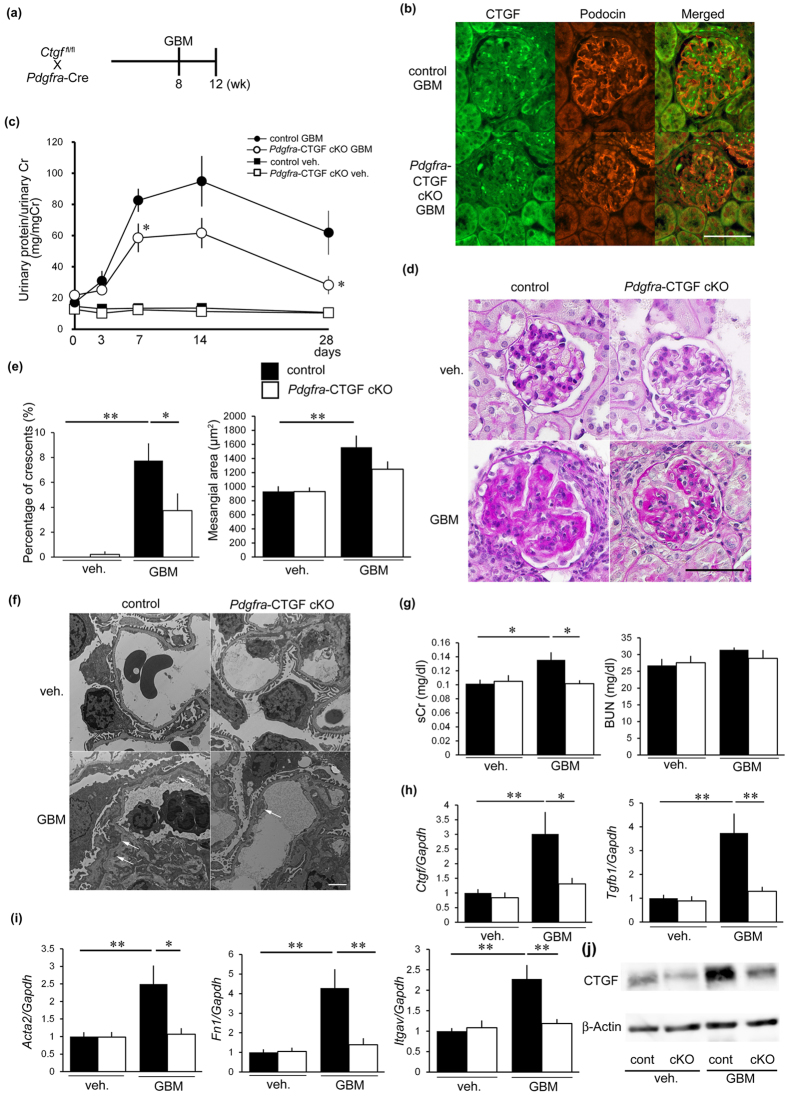
Reduction of CTGF in mesangial cell ameliorated glomerulonephritis. (**a**) Experimental protocol anti-GBM nephritis in *Pdgfra*-CTGF cKO mice. Anti-GBM nephritis was induced at 8 weeks of age. Mice were killed at 4 weeks after induction of anti-GBM nephritis. (**b**) Double immunofluorescence staining for CTGF (green) and podocin (red) in the glomeruli of *Pdgfra*-CTGF cKO or control mice at 4 weeks after induction of anti-GBM nephritis. Bar represents 50 μm. (**c**) Changes in proteinuria during the course of the anti-GBM nephritis. Closed squares, vehicle-treated control mice; open squares, vehicle-treated *Pdgfra*-CTGF cKO mice; closed circles, control mice with nephritis; open circles, *Pdgfra*-CTGF cKO mice with nephritis. (**d**) Representative photomicrographs of the kidneys at 4 weeks after induction of anti-GBM nephritis (PAS staining). Left upper panel, vehicle-treated control mice; right upper panel, vehicle-treated *Pdgfra*-CTGF cKO mice; left lower panel, control mice with anti-GBM nephritis; right lower panel, *Pdgfra*-CTGF cKO mice with anti-GBM nephritis. Bar represents 50 μm. (**e**) The percentage of crescent formation (left panel) and the mesangial area (right panel) in anti-GBM nephritis. (**f**) Electron microscopic analysis of glomeruli in anti-GBM nephritis in *Pdgfra*-CTGF cKO mice. Dense deposit accumulation along GBM was reduced in *Pdgfra*-CTGF cKO mice with nephritis (white arrows). Bar represents 2,000 nm. (**g**) Serum creatinine and BUN levels in anti-GBM nephritis in *Pdgfra*-CTGF cKO mice. (**h**) Expression of *Ctgf* and *Tgfb1* mRNA in glomeruli at 4 weeks after induction of anti-GBM nephritis. (**i**) Expression of *Acta2, Fn1* and *Itgav* mRNA in glomeruli. (**j**) Representative image of glomerular CTGF protein by Western blotting. Immunoblot of β-actin was used as control. Full-length blots are presented in Supplementary Figure S12. Values were expressed as means ± s.e. ^*^P < 0.05, ^**^P < 0.01 vs control mice with anti-GBM nephritis. Vehicle-treated control mice (n = 6), vehicle-treated *Pdgfra*-CTGF cKO mice (n = 6), control mice with anti-GBM nephritis (n = 9), *Pdgfra-*CTGF cKO mice with anti-GBM nephritis (n = 6). Veh, vehicle; GBM, anti-GBM nephritis.

To examine a role of CTGF expressed by mesangial cells in anti-GBM nephritis, we induced anti-GBM nephritis in *Pdgfra*-CTGF cKO mice (Fig. 4a). CTGF was expressed by cells other than podocytes, presumably mesangial cells, in nephritis (Fig. 4b). *Pdgfra*-CTGF cKO mice with nephritis showed reduction of CTGF expression in mesangial cells (Fig. 4b) and proteinuria at 1 and 4 weeks (Fig. 4c)

Crescent formation was significantly reduced in *Pdgfra*-CTGF cKO mice with nephritis and mesangial expansion tended to be decreased in *Pdgfra*-CTGF cKO mice with nephritis compared with control mice with nephritis (Fig. 4d,e). Electron microscopic analysis revealed a reduction in dense deposit accumulation along the GBM in *Pdgfra*-CTGF cKO mice with nephritis (Fig. 4f). *Pdgfra*-CTGF cKO mice exhibited a reduction in the serum creatinine level, but not in BUN levels compared with control mice with nephritis (Fig. 4g). Glomerular *Ctgf* expression was decreased by 60% in *Pdgfra*-CTGF cKO mice with nephritis compared with control mice with nephritis (Fig. 4h). *Tgfb1, Acta2, Fn1* and *Itgav* mRNA expression in the glomeruli was increased in control mice with nephritis, and this increase was reduced in *Pdgfra*-CTGF cKO mice with nephritis (Fig. 4h,i). These results indicate that deletion of mesangial cell-derived CTGF ameliorates glomerular injury in anti-GBM nephritis.

### Role of macrophages in anti-GBM nephritis

**Figure 5:**
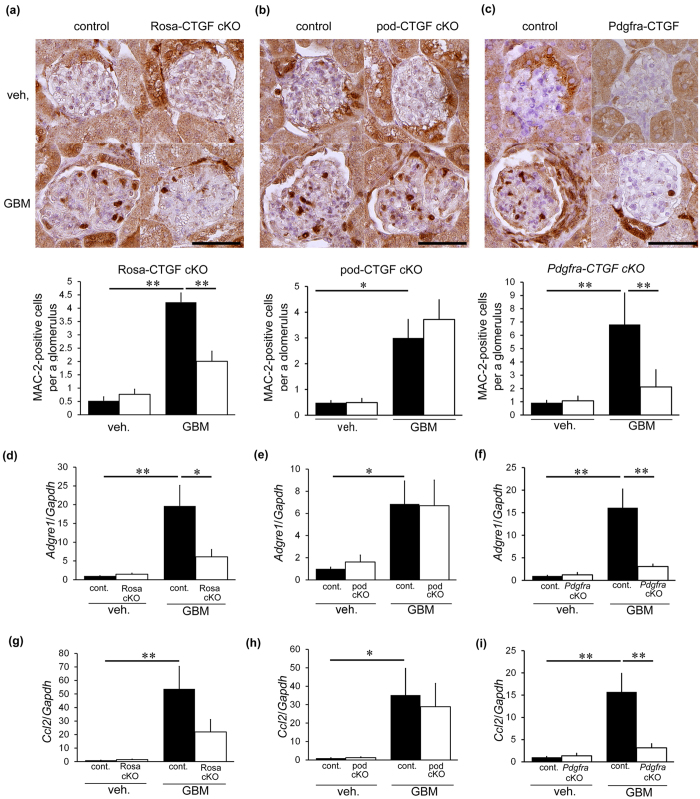
Role of CTGF on macrophage accumulation in anti-GBM nephritis. (**a–c**) Immunohistochemical studies for MAC-2 to analyze the number of MAC-2-positive cells per a glomerulus of Rosa-CTGF cKO mice (**a**), pod-CTGF cKO mice (**b**) and *Pdgfra*-CTGF cKO mice (**c**) at 4 weeks after induction of anti-GBM nephritis. Bar represents 50 μm. (**d–f**) Expression of *Adgre1* (F4/80) mRNA in the glomeruli of Rosa-CTGF cKO mice (**d**), pod-CTGF cKO mice (**e**) and *Pdgfra*-CTGF cKO mice (**f**) at 4 weeks after induction of anti-GBM nephritis. (**g–i**) *Ccl2* mRNA expression in glomeruli of Rosa-CTGF cKO mice (**g**), pod-CTGF cKO mice (**h**) and *Pdgfra*-CTGF cKO mice (**i**) at 4 weeks after induction of anti-GBM nephritis. Veh., vehicle; GBM, anti-GBM nephritis; Rosa cKO, Rosa-CTGF cKO mice; pod cKO, pod-CTGF cKO mice; *Pdgfra* cKO, *Pdgfra*-CTGF cKO mice. Values were expressed as the means ± s.e. ^*^P < 0.05, ^**^P < 0.01.

**Figure 6:**
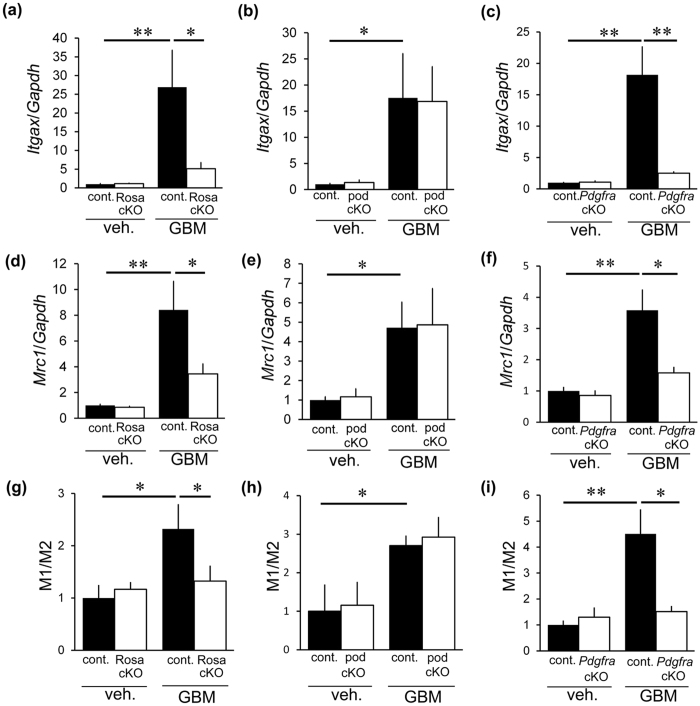
Relevance of CTGF to macrophage phenotypes in anti-GBM nephritis. (**a–c**) Expression of *Itgax* (CD11c, M1 marker) mRNA in the glomeruli of Rosa-CTGF cKO mice (**a**), pod-CTGF cKO mice (**b**) and *Pdgfra*-CTGF cKO mice (**c**) at 4 weeks after induction of anti-GBM nephritis. (**d–f**) *Mrc1* (CD206, M2 marker) mRNA expression in the glomeruli of Rosa-CTGF cKO mice (**d**), pod-CTGF cKO mice (**e**) and *Pdgfra*-CTGF cKO mice (**f**). (**g–i**) Ratio of mRNA expression of M1/M2 macrophage markers in the glomeruli of Rosa-CTGF cKO mice (**g**), pod-CTGF cKO mice (**h**) and *Pdgfra*-CTGF cKO mice (**i**).Veh, vehicle; GBM, anti-GBM nephritis; Rosa cKO, Rosa-CTGF cKO mice; pod cKO, pod-CTGF cKO mice; *Pdgfra* cKO, *Pdgfra*-CTGF cKO mice. Values were expressed as the means ± s.e. ^*^P < 0.05, ^**^P < 0.01.

We evaluated glomerular MAC-2-positive macrophages in each CTGF cKO mouse line because macrophages have been shown to aggravate anti-GBM nephritis^17^,^18^,^19^. Mac-2, also known as galectin-3, is positive for macrophages and distal tubular cells in the kidney^20^,^21^, thus positive cells within glomerular area indicate macrophages. Control mice with nephritis showed an increase in MAC-2-positive cells (Fig. 5a). Rosa-CTGF cKO mice with nephritis exhibited significantly less accumulation of glomerular MAC-2-positive cells (Fig. 5a). In contrast, pod-CTGF cKO mice with nephritis showed no decrease in the number of MAC-2-positive cells in glomeruli (Fig. 5b). Accumulation of MAC-2-positive cells in *Pdgfra*-CTGF cKO mice with nephritis was significantly milder than that in control mice with nephritis (Fig. 5c), indicating that mesangial cells play an important role in macrophage infiltration. Gene expression of a macrophage marker F4/80 (*Adgre1*) and CCL2, which has a potent activity to regulate migration and infiltration of macrophages^22^, showed similar results (Fig. 5d–i). To study M1 and M2 macrophage subtypes (or polarity) in glomeruli with and without anti-GBM nephritis, the ratio of CD11c (*Itgax*, M1 marker) and CD206 (*Mrc1*, M2 marker) was examined (Fig. 6a–f). The M1/M2 ratio in control mice with anti-GBM nephritis was higher than control mice without nephritis. M1/M2 ratios were significantly lower in Rosa-CTGF cKO and *Pdgfra*-CTGF cKO mice with nephritis compared with those in control mice with nephritis, but not in pod-CTGF cKO mice with nephritis (Fig. 6g–i).

### Macrophage recruitment in Rosa-CTGF cKO mice with anti-GBM nephritis at the earlier stage

We examined the effects of CTGF deletion at the earlier stage, 1 week. Rosa-CTGF cKO mice or control mice were administered with anti-GBM serum (Supplementary Fig. 6a), and renal examination was conducted at 1 week later. Induction of anti-GBM nephritis resulted in increase of CTGF protein expression at 1 week predominantly in the glomeruli, and Rosa-CTGF cKO mice with anti-GBM nephritis exhibited a decrease in CTGF expression by podocytes and mesangial cells (Supplementary Fig. 6b). Rosa-CTGF cKO mice with anti-GBM nephritis exhibited significantly reduced proteinuria at 1 week compared with control mice with nephritis (Supplementary Fig. 6c; *P* < 0.05). Microscopic examination indicated increased crescent formation and mesangial expansion were significantly ameliorated in Rosa-CTGF cKO mice with nephritis as follows (Supplementary Fig. 6d,e). The number of macrophages in glomeruli was evaluated by immunohistochemistry for MAC-2 (Supplementary Fig. 6f). Rosa-CTGF cKO mice with nephritis exhibited significantly less accumulation of glomerular MAC-2-positive cells compared with control mice with nephritis (Supplementary Fig. 6g). Serum creatinine levels were elevated in control mice with nephritis; however, no significant difference was observed between control and Rosa-CTGF cKO mice with nephritis (Supplementary Fig. 6h). Electron microscopic analysis demonstrated both control and Rosa-CTGF cKO mice with nephritis demonstrated slight dense deposit accumulation (Supplementary Fig. S7a).

Glomerular *Ctgf* expression decreased in Rosa-CTGF cKO mice with nephritis compared with control mice with nephritis (Supplementary Fig. S7b). Glomerular gene expression of integrin αv (*Itgav*) mRNA expression was increased in control mice with nephritis and this increase was reduced in Rosa-CTGF cKO mice with nephritis at 1 week (Supplementary Fig. S7c). Expression of *Fn1, Acta2, Tgfb1, Adgre1* or *Ccl2* was tended to be reduced in Rosa-CTGF cKO mice with nephritis, but not significant (Supplementary Fig. S7d–h). We also examined M1 and M2 macrophage subtypes at 1 week in glomeruli with and without anti-nephritis. Expression of both CD11c (*Itgax*) and CD206 (*Mrc1*) was increased in control mice with nephritis, and their expression was significantly reduced in Rosa CTGF cKO mice with nephritis (Supplementary Fig. S7i,j). When M1/M2 ratio was examined, no significant difference was observed among control and Rosa-CTGF cKO with nephritis at 1 week (Supplementary Fig. S7k). These results suggest that systemic deletion of CTGF ameliorates proteinuria and glomerular injury by reducing macrophage recruitment at 1 week after induction of anti-GBM nephritis.

### Effects of CTGF inhibition in cultured mesangial cells and macrophages

**Figure 7:**
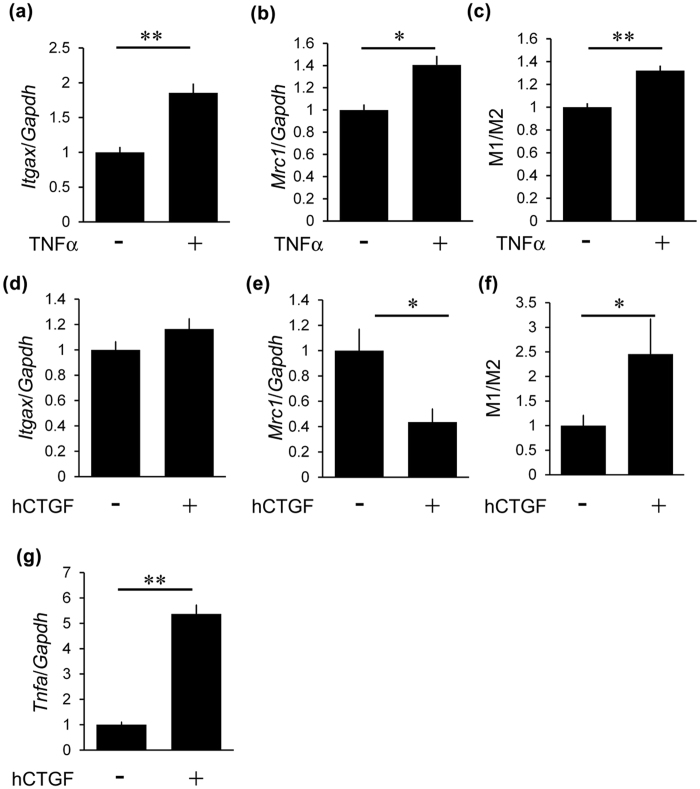
The effects of CTGF on macrophage subtypes. (**a,b**) Expression of *Itgax* (**a**) and *Mrc1* (**b**) by 10 ng/ml TNF-α in RAW264.7 cells at 3 h after stimulation. (**c**) Ratio of mRNA expression of M1/M2 macrophage markers in RAW264.7 cells after stimulation with TNF-α. (**d,e**) Expression of *Itgax* (**d**) and *Mrc1* (**e**) by 1000 ng/ml CTGF in RAW264.7 cells. (**f**) Ratio of mRNA expression of M1/M2 macrophage markers in RAW264.7 cells after stimulation with 1000 ng/ml CTGF. (**g**) Induction of *Tnfa* expression by treatment with 1000 ng/ml CTGF in RAW264.7 cells. hCTGF, recombinant human CTGF. n = 6, each. Values were expressed as the means ± s.e. ^*^P < 0.05, ^**^P < 0.01.

Next, we focused on the effect of CTGF on inflammation and macrophage subtypes *in vitro*. We examined whether TNF-α induced macrophage subtype. Stimulation with TNF-α increased both *Itgax* and *Mrc1* mRNA in cultured macrophages (Fig. 7a,b), leading to the increment of M1/M2 ratio (Fig. 7c). Next, we examined the effects of CTGF on macrophage subtypes. Although expression of *Itgax* was not altered by the stimulation of CTGF (Fig. 7d), expression of *Mrc1* was significantly decreased with CTGF in cultured macrophages (Fig. 7e). Consequently, the M1/M2 ratio was significantly upregulated with the stimulation of recombinant CTGF (Fig. 7f). In addition, CTGF induced *Tnfa* mRNA in cultured macrophages (Fig. 7g).

**Figure 8:**
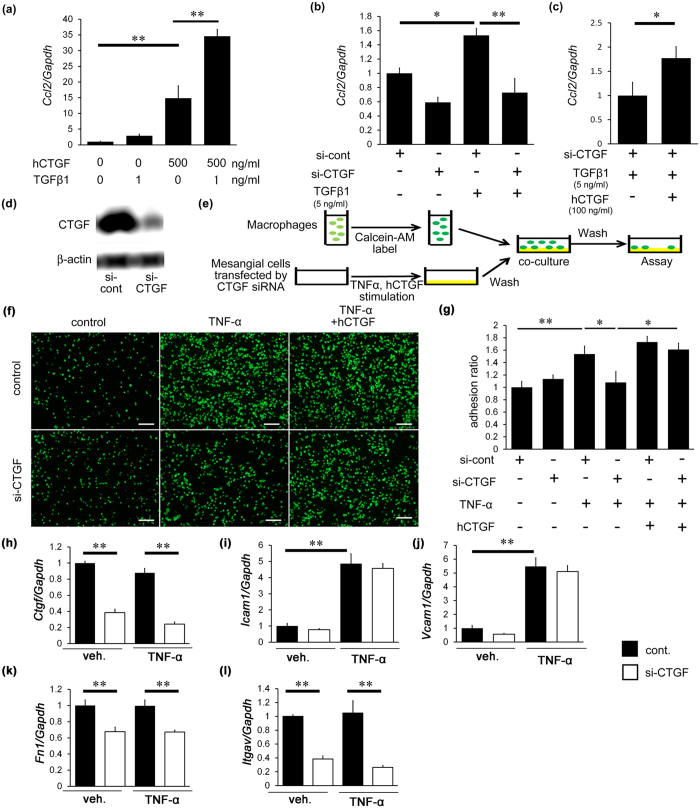
The effects of CTGF on chemotasis in cultured mesangial cells and adhesion of cultured macrophage to activated mesangial cells. (**a**) Induction of *Ccl2* expression by treatment with 500 ng/ml CTGF and/or 1 ng/ml TGF-β1 in cultured mesangial cells. (**b**) Reduction of *Ccl2* expression by CTGF siRNA in cultured mesangial cells. (**c**) Amelioration of siRNA-mediated *Ccl2* reduction by treatment of exogenous CTGF (100 ng/ml). hCTGF, recombinant human CTGF; siCTGF, siRNA against CTGF. (**d**) Immunoblt of CTGF by CTGF siRNA in cultured mesangial cells by Western blotting. Full-length blots are presented in Supplementary Figure S13. (**e–g**) Macrophage adhesion to cultured mesangial cells on culture plates. (**e**) Experimental protocol of adhesion of fluorescein-dye-labeled RAW264.7 cells to 10 ng/ml TNF-α-stimulated mesangial cells. (**f**) Inhibition of CTGF by siRNA reduced macrophage adhesion, and this reduction was ameliorated by exogenous human CTGF (100 ng/ml). Bar represents 100 μm. (**g**) Quantitative analyses of adhesion of RAW264.7 cells to cultured mesangial cells. (**h**) *Ctgf* mRNA expression in cultured mesangial cells with the stimulation of 10 ng/ml TNF-α. (**i–l**) Expression of *Icam1* (**i**), *Vcam1* (**j**), *Fn1* (**k**) and *Itgav* (**l**) mRNA in mesangial cells after stimulation of 10 ng/ml TNF-α. n = 6, each. ^*^P < 0.05, ^**^P < 0.01.

To examine the effect of CTGF on migration, we first investigated the expression of *Ccl2* following treatment with CTGF and/or TGF-β1 in cultured mesangial cells, because CCL2 is a major mediator of macrophage recruitment. CTGF and TGF-β1 upregulated *Ccl2* mRNA expression, and the combination of CTGF and TGF-β1 further enhanced *Ccl2* expression in cultured mesangial cells (Fig. 8a). The expression of *Ccl2* was reduced by CTGF inhibition in TGF-β1-treated mesangial cells (Fig. 8b). Treatment with recombinant human CTGF overcame the effects of endogenous CTGF inhibition in TGF-β1-induced *CCl2* mRNA upregulation (Fig. 8c). Inhibition of CTGF by si-CTGF was confirmed by Western blotting (Fig. 8d). These results suggest that CTGF upregulation in mesangial cells plays an important role in regulating *Ccl2* expression.

We further investigated the effects of CTGF on adhesion of macrophages to activated mesangial cells (Fig. 8e). Fluorescein-dye-labeled RAW264.7 cells were co-cultured with recombinant TNF-α-stimulated mesangial cells on culture plates (Fig. 8f,g). The increase in macrophage adhesion by TNF-α stimulation was significantly inhibited by CTGF knockdown in mesangial cells, and this reduction was canceled by exogenous CTGF administration (Fig. 8f,g). We next examined the mRNA expression of adhesion molecules in cultured mesangial cells relevant to macrophage adhesion. The expression of *Icam1* and *Vcam1* did not change following CTGF knockdown but was increased by TNF-α stimulation (Fig. 8h–j). In contrast, the expression of *Fn1* and *Itgav* was significantly reduced by CTGF knockdown; however this reduction was not affected by TNF-α (Fig. 8k,l), raising a possibility that CTGF may enhance macrophage adhesion to mesangial cells through expression of fibronectin and integrin αv. We next examined the role of CTGF on macrophage adhesion to endothelial cells (Supplementary Fig. S8a). The increase of macrophage adhesion to TNF-α-stimulated HUVEC was significantly attenuated by CTGF reduction (Supplementary Fig. S8b,c), indicating that CTGF is involved in macrophage adhesion to endothelial cells.

## Discussion

In the present study, we investigated a role of CTGF in a mouse model of anti-GBM nephritis, using three types of conditional CTGF KO mice. Although a previous study showed that inducible systemic CTGF KO mice lacking exon 4 grow normally^23^, the present study is the first to describe a role of CTGF in kidney diseases using systemic conditional KO mice. Our work revealed that renal injury induced by anti-GBM nephritis is markedly inhibited in inducible systemic CTGF KO mice lacking the whole CTGF coding region. Blockade of CTGF has been shown to exert renoprotective effects in a number of renal diseases, including diabetic nephropathy and unilateral ureteral obstruction^15^,^24^. This study clearly showed that inhibition of CTGF can ameliorate immune-complex mediated glomerulonephritis. We demonstrated the reduction of urinary protein, crescentic formation, mesangial expansion and electron dense deposits without changing heterologous, autologous antibodies or C3 complement deposition. The previous reports showed that discrepancy between IgG deposition and electron dense deposits in anti-GBM nephritis was documented^25^,^26^, which were consistent with our results.

Although we demonstrated the importance of CTGF on anti-GBM nephritis, identification of the specific glomerular cell types responsible for pathological CTGF expression has remained unanswered. To address this issue, we employed other conditional KO mice in anti-GBM nephritis. Previous reports revealed that podocytes are the cells most severely affected by anti-GBM nephritis^27^, demonstrating the importance of podocytes in this model. In this context, we investigated whether CTGF reduction in podocytes could ameliorate anti-GBM nephritis using pod-CTGF cKO mice, because CTGF has been shown to be upregulated by podocytes and mesangial cells in crescentic glomerulonephritis in both humans and rats^13^,^28^. In crescentic glomerulonephritis, podocytes are considered to contribute to crescent formation and proteinuria^29^. Surprisingly, the present study demonstrated that pod-CTGF cKO mice with anti-GBM nephritis showed no improvement in proteinuria or histological changes. These results prove that abundant CTGF expression in podocytes does not play a major role in glomerular injury of anti-GBM nephritis.

Concerning the glomerular cell types that contribute to CTGF actions in nephritis, we next focused on mesangial cells. However, there are no known mice strains that specifically express Cre in mesangial cells^30^. Therefore, we focused on PDGFRα, which is weakly expressed predominantly in mesangial cells under normal conditions and more strongly in glomerulonephritis^31^,^32^. We confirmed that PDGFRα was expressed in mesangial regions in mice with anti-GBM nephritis (Supplementary Fig. S4b), and confirmed minor contribution of bone marrow-derived cells (Supplementary Fig. S5). Notably, *Pdgfra*-CTGF cKO mice with anti-GBM nephritis exhibited significantly reduced proteinuria and decreased crescent formation and mesangial expansion, similar to findings observed in Rosa-CTGF cKO mice. Of note, the creatinine levels only in *Pdgfra*-CTGF cKO mice with anti-GBM nephritis was reduced compared with that in control mice with nephritis, although Rosa-CTGF cKO with nephritis mice did not show the decreased creatinine level. These results may be due to incomplete deletion of CTGF alleles in *Pdgfra*-positive cells of Rosa-CTGF cKO mice. Although we and others have shown that PDGFRα is also expressed in the collecting ducts, proximal tubular cells and interstitial fibroblasts of the kidney^33^, glomerular mesangial cells expressing both PDGFRα and CTGF appear to make predominant contribution to glomerular injury in anti-GBM nephritis. It is a limitation of this study that *Pdgfra*-Cre mice express Cre recombinase not only in mesangial cells but also in other type cells.

CTGF has been reported to be a crucial mediator of organ fibrosis and proinflammatory responses. CTGF has been also shown to upregulate chemokines and adhesion molecules to promote cell migration and adhesion^34^,^35^,^36^,^37^. In the present study, accumulation of MAC-2-positive cells in the glomeruli was decreased in Rosa-CTGF cKO and *Pdgfra*-CTGF cKO mice at 4weeks, but not in pod-CTGF cKO mice. The glomerular *Ccl2* and *Adgre1* expression and the M1/M2 macrophage marker ratio were decreased in Rosa-CTGF cKO and *Pdgfra*-CTGF cKO mice, indicating that CTGF expression particularly in mesangial cells is critical to induction of inflammation in anti-GBM nephritis. Sánchez-López *et al*. demonstrated that systemic administration of full length or C-terminal module 4 of CTGF to mice increase renal expression of *Ccl2*, leading to recruitment of immune cells^35^,^38^. Several studies have reported that the inhibition of macrophage infiltration ameliorates anti-GBM nephritis^17^,^18^,^19^. We examined the renal changes at 1 week after induction of nephritis using Rosa-CTGF cKO mice, indicating that CTGF deletion resulted in reduced accumulation of macrophages. Recently, CTGF has been shown to be involved with increase of M1 and decrease of M2 macrophages in pancreatic β-cell ablation model^39^. Our study reveals that ablation of CTGF did not reduce M1/M2 ratio at 1 week but at 4 weeks *in vivo* and that recombinant CTGF increased M1/M2 ratio *in vitro*. Further studies are necessary to elucidate the mechanism of macrophage subtype transition by CTGF at each time point. We investigated the effects of CTGF reduction on inflammation in cultured mesangial cells *in vitro*, demonstrating that mRNA expression of *Ccl2* was increased by CTGF stimulation. TGF-β-induced *Ccl2* expression was downregulated by CTGF inhibition and this downregulation was abrogated by exogenous CTGF. These data suggest that CTGF from mesangial cells, not podocytes, may be required for the upregulation of *Ccl2* expression not only in anti-GBM nephritis but also in other types of glomerulonephritis such as IgA nephropathy or crescentic glomerulonephritis, because CTGF expression and accumulation of macrophages in mesangial area are documented in these glomerular diseases.

The present study demonstrated that increased macrophage adhesion to mesangial cells in response to TNF-α was significantly reduced by CTGF knockdown using siRNA transfection. Although previous reports have demonstrated that CTGF enhances adhesion through interactions with integrins and fibronectin on endothelial cells and fibroblasts^40^,^41^, we demonstrate here the importance of mesangial cells in regulating macrophage adhesion through CTGF-dependent expression of fibronectin and integrin α_v_ during nephritis. As previous reports showed that endothelial cells are involved in the macrophage chemotaxis and attachments, this study also evaluated the important role of CTGF on macrophage adhesion to endothelial cells. Further analysis will be necessary to reveal a precise mechanism playing a role in macrophage adhesion on endothelial cells. These results of the present study suggest that CTGF induces macrophage accumulation in anti-GBM nephritis by enhancing both chemotaxis and adhesion, and that reduction of CTGF, particularly in mesangial cells, ameliorates nephritis via inhibition of macrophage infiltration (Supplementary Fig. S9). The present study unveils a novel crosstalk between mesangial cells and macrophages in the progression of glomerulonephritis^42^.

In conclusion, systemic reduction in CTGF expression can ameliorate proteinuria and glomerular injury in anti-GBM nephritis through suppression of inflammation and extracellular matrix accumulation. Furthermore, we observed a novel role of mesangial cells in enhancing macrophage chemotaxis and adhesion in a CTGF-dependent manner. These findings demonstrate the importance of CTGF in the progression of glomerulonephritis and suggest that CTGF represents a promising therapeutic target for glomerulonephritis.

## Materials and Methods

### Experimental animals

A BAC clone containing the complete mouse *Ctgf* gene was isolated from a mouse C57BL/6 J genomic library. A DNA fragment containing *Ctgf* gene was generated by PCR using the BAC clone and then inserted into the targeting vector. Two *loxP* sites were introduced into the targeting vector at 12 bp upstream of the ATG codon and immediately after the stop codon TAA by PCR based mutagenesis^43^. The targeting vector contained two flippase recombination target (FRT) sites that flank the neomycin resistant gene. The targeting vector was designed to induce conditional deletion of exons 1–5 of the mouse *Ctgf* gene flanked with loxP sequences (Supplementary Fig. S1a). The final linearized vector was introduced into mouse C57BL/6 J embryonic stem (ES) cells and these cells were selected with Geneticin (G418, ThermoFisher Scientific, Waltham, MA) and Fialuridine (FIAU, Sigma Aldrich, St. Louis, MO). Positive clones were selected by Southern blotting and PCR analysis, injected into blastocysts, and brought to term in surrogate mothers. These animals were crossed with transgenic mice expressing flippase constitutively to remove the neomycin resistance gene.

C57BL/6-Gt (ROSA) 26Sortm9^*(cre/ESR1) Art*^ (ROSA26-CreER^T2^) mice^44^ were purchased from ARTEMIS Pharmaceuticals. C57BL/6-Tg(*Pdgfra*-Cre)1Clc/J mice (*Pdgfra*-Cre mice) and B6.Cg-Gt(*ROSA*)Sor^tm6(CAG-ZsGreen1)Hze^/J mice were purchased from the Jackson Laboratory. *Nephrin-Cre* mice were constructed as described previously^45^. Tamoxifen-inducible systemic CTGF conditional KO (Rosa-CTGF cKO) mice were generated by mating *CTGF*^*fl/fl*^ mice and Rosa-CreER^T2^ mice. Three-week-old ROSA26-CreER^T2^; *Ctgf*^*fl/fl*^ mice or control [*CTGF*^*fl/fl*^, Cre (−)] mice were intraperitoneally injected with 0.05 mg/gBW 4-hydroxytamoxifen (4-OHT, Sigma Aldrich) for 3 days. Pod-CTGF cKO and *Pdgfra*-CTGF cKO mice were generated by crossing *CTGF*^*fl/fl*^ mice with *Nephrin-Cre* or *Pdgfra-Cre* mice, respectively. B6.Cg-Gt (*ROSA*) Sortm6 ^(CAG-ZsGreen1) Hze^/J mice were crossed with *Pdgfra-Cre* mice to generate *Pdgfra*-ZsGreen mice. Mice were maintained in a specific pathogen-free facility. All animal experiments were performed in accordance with Fundamental Guidelines for Proper Conduct of Animal Experiment and Related Activities in Academic Research Institutions and were approved by the Animal Experimentation Committee of Kyoto University Graduate School of Medicine.

### Induction of anti-glomerular basement antibody nephritis

An accelerated form of anti-GBM nephritis was induced in Rosa-CTGF cKO, pod-CTGF cKO, *Pdgfra*-CTGF cKO, and *Pdgfra*-ZsGreen mice. Preparation of anti-GBM antiserum in rabbits was performed as described previously^46^. Mice were immunized by an intraperitoneal injection of 0.5 mg/20 gBW of normal rabbit IgG (MP Biomedicals, Santa Ana, CA) emulsified with complete Freund’s adjuvant (Difco, Detroit, MI) at 8 weeks of age, and anti-GBM antibody or vehicle (normal rabbit serum) was injected into the tail vein 5 days later. Urinary protein levels and body weights were measured on days 0, 3, 7, 14, and 28 after induction of anti-GBM nephritis. For urinary measurements, each animal was housed separately in a metabolic cage (Shinano Manufacturing, Tokyo, Japan). Urinary protein, urinary creatinine, serum creatinine and BUN were measured by enzymatic method (SRL, Tokyo, Japan). Mice were killed at 1 or 4 weeks after the induction of anti-GBM nephritis under pentobarbital anesthesia. Blood and kidney samples were collected for biochemical and histological analyses. Glomeruli were isolated by the graded sieving method as described previously^43^.

### Renal histology and electron microscopy

Histological and electron microscopic examinations were performed as described previously^14^,^47^. Briefly, kidney sections stained with periodic acid-Schiff (PAS) and Masson’s trichrome (MT) were examined by light microscopy (IX-81, Olympus, Tokyo, Japan). The ratio of crescentic glomeruli to total glomeruli was calculated as the crescent formation ratio. Mesangial areas in 10 superficial glomeruli were measured quantitatively using computer-aided manipulator (MetaMorph software, Molecular Devices, Sunnyvale, CA) with mean values calculated. These procedures were performed by investigators blinded to the origin of the slides. Electron microscopic examination was performed in an electron microscope (H-7600, Hitachi, Tokyo, Japan).

### Immunohistochemistry

Immunofluorescence and immunohistochemical studies of the kidney samples were performed as previously described^47^. Primary antibodies used were FITC-labeled donkey anti-rabbit IgG (Jackson ImmunoResearch, West Grove, PA), FITC-labeled anti-mouse IgG (Jackson ImmunoResearch), FITC-labeled goat anti-mouse C3 antibodies (MP Biomedicals), goat anti-CTGF antibody (Santa Cruz Biotechnology Inc., Santa Cruz, CA), rabbit anti-podocin antibody (Sigma-Aldrich), rat anti-MAC-2 antibody (Cedarlane, Burlington, Ontario, Canada), rat anti-mouse PECAM1 antibody (BD Biosciences, San Jose, CA), rat anti-mouse PDGFRβ antibody (Biolegend, San Diego, CA), goat anti-Megalin antibody (Santa Cruz Biotechnology), and goat anti-AQP2 (Santa Cruz Biotechnology). Four-μm-thick cryostat sections fixed in acetone were incubated with primary antibodies and then examined by fluorescence microscopy. For double immunofluorescence analysis of CTGF and podocin, the kidney sections were fixed using Dubosq-Brazil solution and embedded in paraffin. The sections were boiled for 10 min, followed by incubation with goat anti-CTGF antibody and Hylite Flour 555 (Dojindo, Tokyo, Japan)-labeled anti-podocin antibody for 1 hour. The sections were then incubated with FITC-donkey anti goat antibody (Jackson ImmunoResearch). Immunohistochemical analysis of CTGF (Santa Cruz Biotechnology Inc.) and MAC-2 was performed as described previously^15^,^21^. More than 10 consecutive glomerular sections were examined for each mouse, and the mean number of MAC-2-positive cells per glomerular cross-section was calculated. For double immunofluorescence analysis of ZsGreen and podocin, PECAM1, PDGFRβ, Megalin, or AQP2, 10-μm-thick cryostat sections fixed with 4% paraformaldehyde were incubated with Hylite Flour 555-labeled anti-podocin antibody, anti-PECAM1 antibody, anti-PDGFRβ antibody, anti-Megalin antibody, or anti-AQP2 antibody, respectively, for 1 h.

### Cell line and glomerular RNA extraction and real-time RT-PCR

Quantitative real-time RT-PCR was performed using StepOnePlus system (Applied Biosystems, Foster City, CA) as described previously^14^. *Ctgf, Tgfb1, Acta2, Fn1, Itgav, Adgre1, Ccl2, Icam1, Vcam1, Mrc1,* and *Itgax* mRNA expression levels were evaluated. Primers and probes sequences are listed in Supplementary Table 1. Data were normalized to *Gapdh* mRNA (TaqMan rodent *Gapdh* control reagents, Applied Biosystems).

### Western blotting

Western blotting for CTGF and β-actin was performed as described previously^43^.

### Flow cytometry analysis for bone marrow cells

Femurs of *Pdgfra*-ZsGreen mice were harvested and flushed through the ice cold phosphate-buffered saline. Bone marrow cell suspensions were passed through a 35 μm cell strainer to obtain a single cell suspension^48^. Flow cytometric analysis and cell sorting were performed using fluorescence-activated cell sorter Aria II (BD biosciences), following the manufacturer’s instruction^49^.

### Cell culture

A murine mesangial cell line, Mes13, was obtained from American Type Culture Collection (ATCC, Manassas, VA). Mes13 cells were cultured with Dulbecco’s Modified Eagle Medium (DMEM) containing high glucose/Nutrient Mixture F-12 Ham supplemented with 10% fetal bovine serum (FBS). Human umbilical vein endothelial cells (HUVEC) were cultured with EGM^TM^-2 BulletKit^TM^ (Lonza, Basel, Switzerland). An adherent macrophage cell line, Raw 264.7, was obtained from ATCC and were cultured with DMEM containing high glucose concentration with 10% FBS at 37 °C under 5% CO_2_. For the analysis for M1/M2 macrophage subtype transition by CTGF or TNF-α, RAW264.7 cells were stimulated with vehicle or 1000 ng/ml CTGF (PROSPEC, East Brunswick, NJ) in DMEM without FBS, and then harvested 3 h after stimulation. Stimulation with 10 ng/ml recombinant mouse TNF-α (R&D systems, Minneapolis, MA) was performed at the same protocol, and collected at 3 h. To examine mRNA expression of *Ccl2* in mesangial cells, Mes 13 cell were stimulated with 1 ng/ml TGF-β1 and/or 500 ng/ml CTGF, and then harvested 3 h after stimulation.

### Transfections with siRNA

Mes13 cells or HUVEC were transfected with 20 nM siRNA against mouse CTGF (#SI00190974, Qiagen, Germantown, MD) or human CTGF (#SI00029694, Qiagen) using Amaxa Cell Line Nucleofector Kit (Lonza)^43^, and then cultured for 48 h. Negative control siRNA (#SI1022076, Qiagen) was also used. The cells were stimulated with 5 ng/ml TGF-β1 and/or 100 ng/ml recombinant CTGF. Cells were harvested 3 h after stimulation. *Ccl2* mRNA expression was evaluated by TaqMan PCR.

### Adhesion assay

Macrophage-mesangial cell binding assay was performed using the Vybrant cell adhesion assay Kit (Life technologies, Carlsbad, CA) according to the manufacturer’s instruction. Briefly, Mes13 cells or HUVEC transfected with siRNA against CTGF or control were cultured for 24 h in 96-well plates before pretreatment with 10 ng/ml TNF-α. Twenty-four hours after TNF-α stimulation, Mes 13 cells or HUVEC were treated with 100 ng/ml human recombinant CTGF for 3 hours, and then were co-cultured with RAW264.7 cells (5 × 10^6^ cells/ml) which were pretreated with 5 μM calcein-bis (carboxymethyl) aminomethyl (AM) solution in DMEM for 30 min. Two hours after co-culture, cells were washed four times with phosphate-buffered saline to remove non-adherent calcein-AM labeled Raw264.7 cells. Fluorescence intensity was measured using a 2030 ARVO^TM^ X (PerkinElmer, Waltham, MA) at excitation and emission wavelengths of 485 nm and 535 nm, respectively.

### Statistical analysis

Data are expressed as means ± s.e. Statistical analyses were performed using the Student’s t-test, the Tukey-Kramer test, or the Dunnett’s test using JMP9 statistical software (SAS institute, Cary, NC). *P* values less than 0.05 were considered statistically significant.

## Additional Information

**How to cite this article**: Toda, N. *et al*. Crucial Role of Mesangial Cell-derived Connective Tissue Growth Factor in a Mouse Model of Anti-Glomerular Basement Membrane Glomerulonephritis. *Sci. Rep.* **7**, 42114; doi: 10.1038/srep42114 (2017).

**Publisher's note:** Springer Nature remains neutral with regard to jurisdictional claims in published maps and institutional affiliations.

## Supplementary Material

Supplementary Figures and Table
