# Supplementary material for: Crucial Role of Mesangial Cell-derived Connective Tissue Growth Factor in a Mouse Model of Anti-Glomerular Basement Membrane Glomerulonephritis
Source: Sci Rep. 2017 Feb 13;7:42114. doi: 10.1038/srep42114 (PMC5304211; doi:10.1038/srep42114)
Supplement: Supplementary Figures and Table [file srep42114-s1.pdf]

Crucial Role of Mesangial Cell-derived Connective Tissue Growth Factor in a Mouse Model of Anti-Glomerular Basement Membrane Glomerulonephritis

Naohiro Toda[1], Kiyoshi Mori[2,3], Masato Kasahara[4], Akira Ishii[1], Kenichi Koga[1], Shoko Ohno[1], Keita P. Mori[1], Yukiko Kato[1], Keisuke Osaki[1], Takashige Kuwabara[1,5], Katsutoshi Kojima[6], Daisuke Taura[6], Masakatsu Sone[6], Taiji Matsusaka[7], Kazuwa Nakao[8], Masashi Mukoyama[1,5], Motoko Yanagita[1], Hideki Yokoi[1,*]

[1]Department of Nephrology, Graduate School of Medicine, Kyoto University, Kyoto Japan, [2]School of Pharmaceutical Sciences, University of Shizuoka, Shizuoka, Japan, [3]Department of Nephrology and Kidney Research, Shizuoka General Hospital, Shizuoka, Japan, [4]Institute for Clinical and Translational Science, Nara Medical University Hospital, Kashihara, Japan [5]Department of Nephrology, Kumamoto University Graduate School of Medical Sciences, Kumamoto, Japan, [6]Department of Diabetes, Endocrinology and Nutrition, Graduate School of Medicine, Kyoto University, Kyoto, Japan, [7]Department of Molecular Life Sciences, Tokai University School of Medicine, Isehara, Japan, [8]Medical Innovation Center, Graduate School of Medicine, Kyoto University, Kyoto Japan,

[*]**Corresponding author:** Hideki Yokoi, M.D., Ph.D., Department of Nephrology, Graduate School of Medicine, Kyoto University, 54 Shogoin Kawahara-cho, Sakyo-ku, Kyoto 606-8507, Japan.

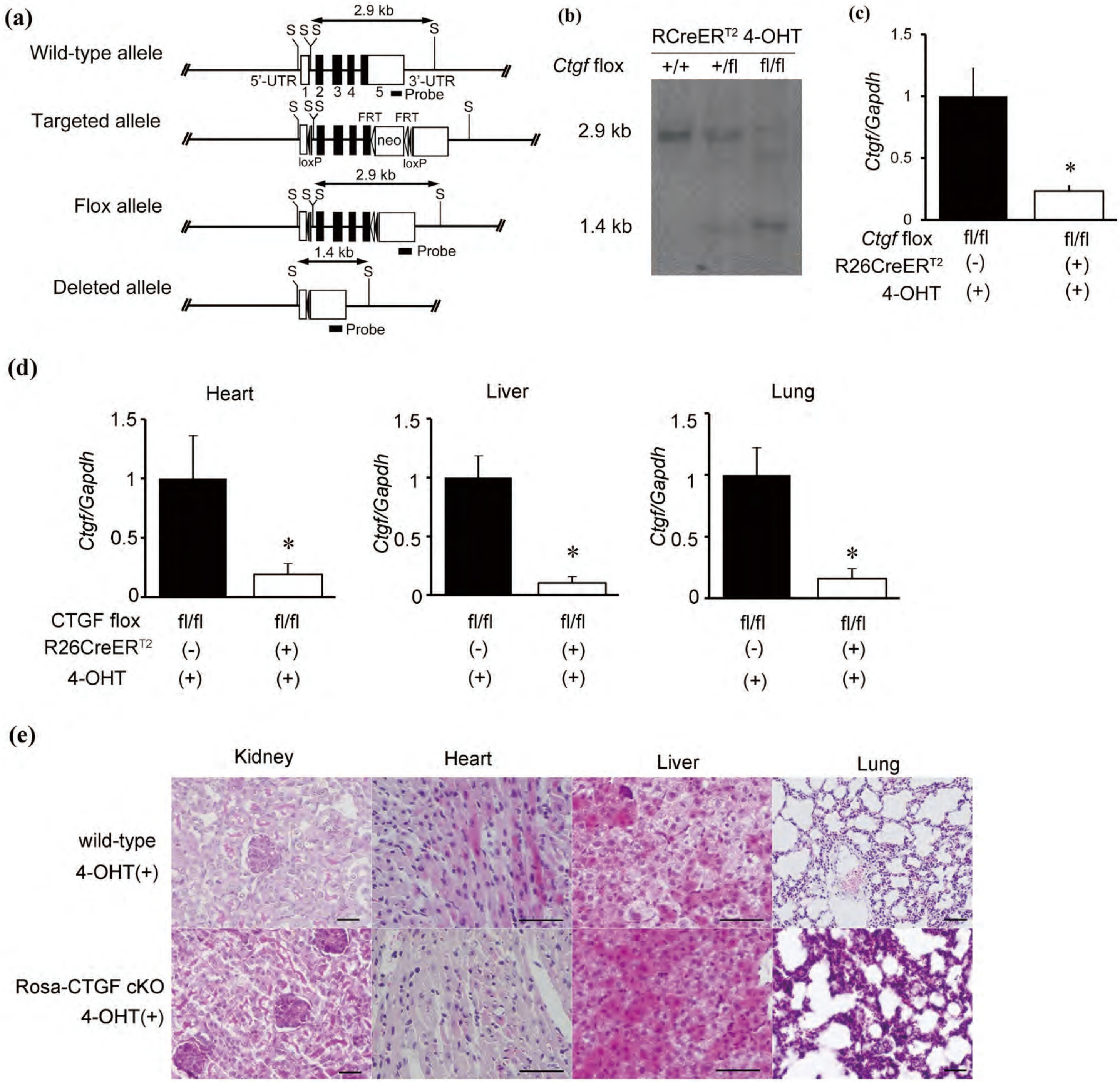

Supplementary Figure S1. Generation of tamoxifen-inducible systemic CTGF conditional KO mice. (a) Schema of targeting strategy. The targeting vector was designed to enable conditional deletion of the entire coding region (closed boxes; exon 1-5) of the mouse *Ctgf* gene. Mouse *Ctgf* gene was flanked by two loxP sites (closed triangles). The neomycin cassette (neo) was flanked by two flippase recognition target (FRT) sites (open triangles). Probes (closed boxes) were used for Southern blotting. S, SmaI restriction sites. (b) Southern blot analysis of *Ctgf* gene by SmaI digestion of DNA extracted from the kidney. Three-week old male mice were treated with 0.05 mg/kgBW 4-OHT for 3 days. The kidneys were obtained at 8 weeks of age. Wild-type allele yielded 2.9-kb band, and deleted allele yielded 1.4-kb band. +, wild-type; fl, floxed. (c and d) Real-time RT-PCR analysis of *Ctgf* mRNA expression in the kidney (c), the heart, the liver and the lung (d). *Gapdh* mRNA expression was used as control. RCreER^{T2}, Rosa26-CreER^{T2}; 4-OHT, 4-hydroxytamoxifen. *P < 0.05. (e) Histological examination of the kidney, heart, liver, and the lung in tamoxifen-inducible CTGF KO mice. 4-OHT, 4-hydroxytamoxifen. Bar represents 50 μm.

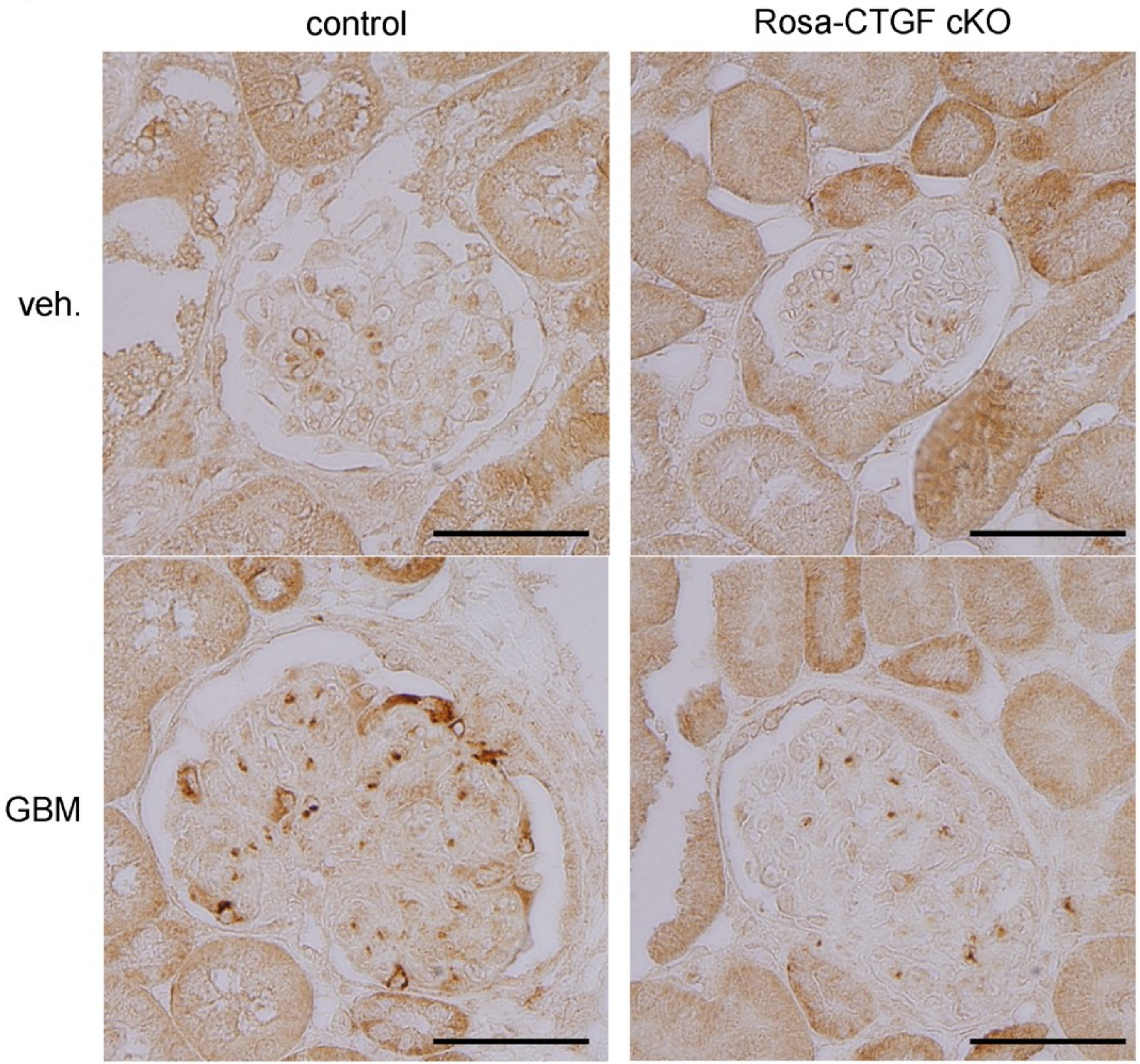

Supplementary Figure S2. Immunohistochemical study for CTGF. Veh., vehicle; GBM, anti-GBM nephritis. Left upper panel, vehicle-treated control mice; right upper panel, vehicle-treated Rosa-CTGF cKO mice; left lower panel, control mice with anti-GBM nephritis; right lower panel, Rosa-CTGF cKO mice with anti-GBM nephritis. Bar represents 50 μm.

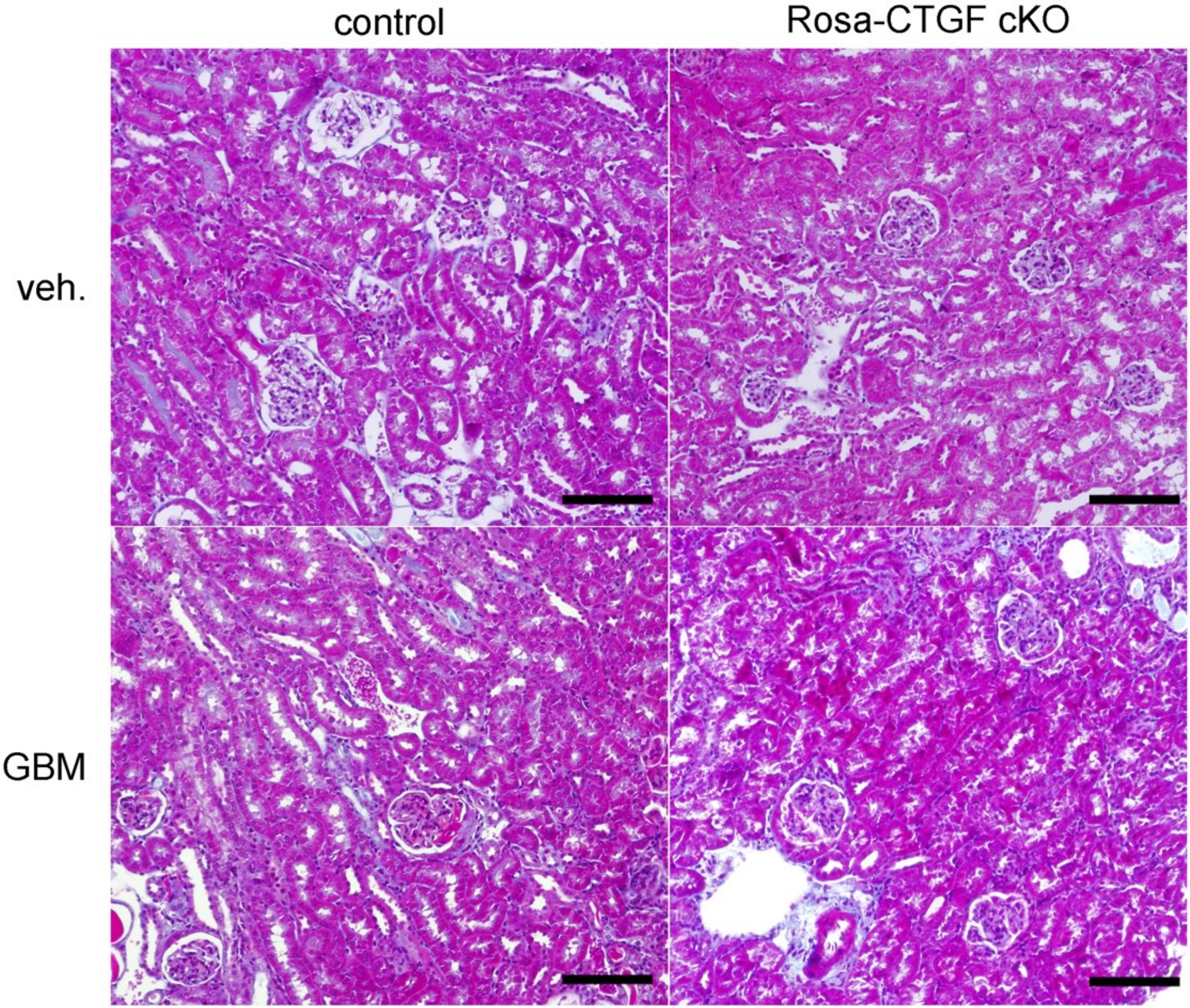

Supplementary Figure S3. Mild tubulointerstitial fibrosis after induction of anti-GBM nephritis in Rosa-CTGF cKO mice. Representative photomicrographs of the kidneys at 4 weeks after induction of anti-GBM nephritis of the kidney (MT staining). Veh., vehicle; GBM, anti-GBM nephritis. Left upper panel, vehicle-treated control mice; right upper panel, vehicle-treated Rosa-CTGF cKO mice; left lower panel, control mice with anti-GBM nephritis; right lower panel, Rosa-CTGF cKO mice with anti-GBM nephritis. Bar represents 100 μm.

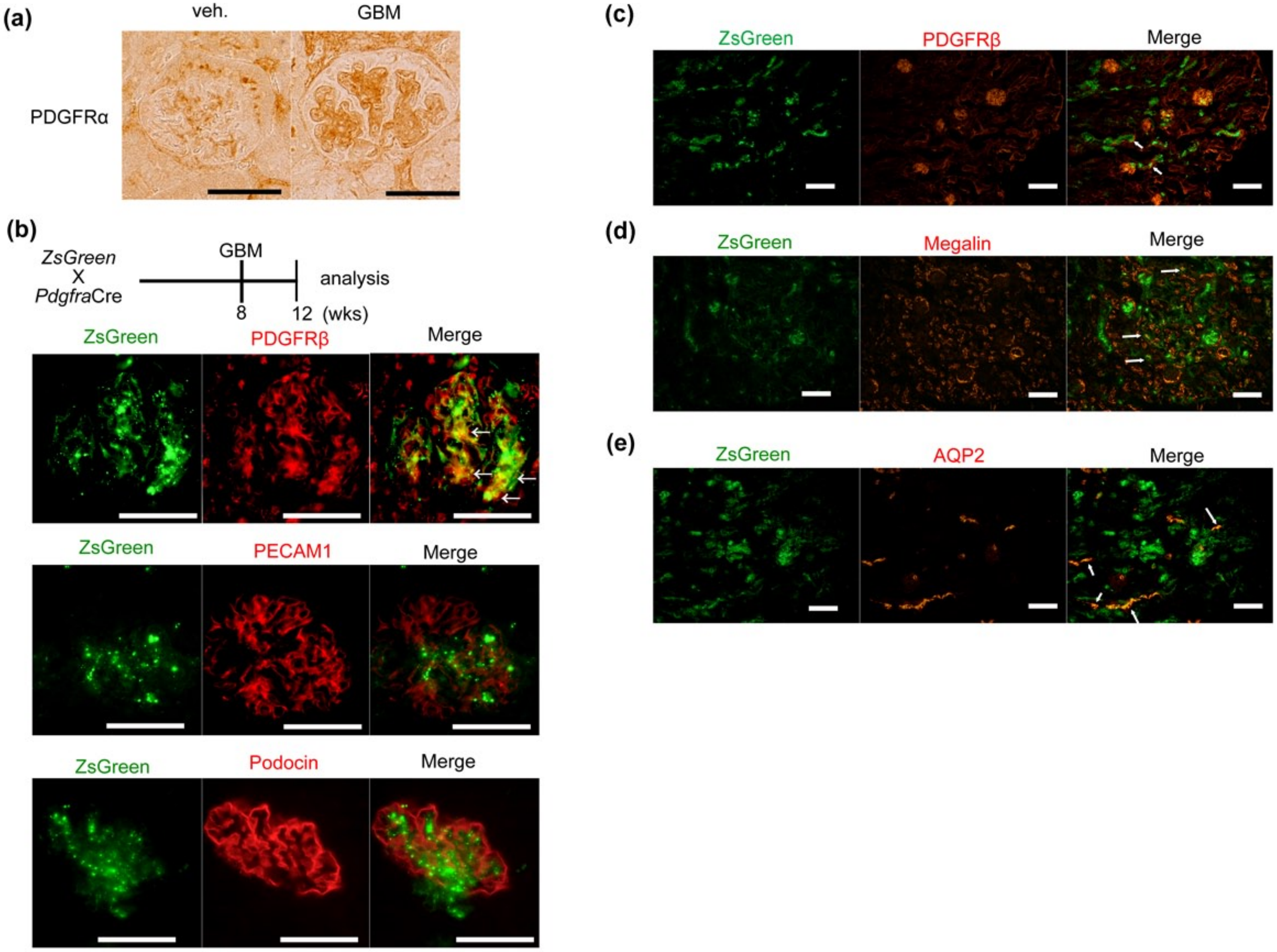

Supplementary Figure S4. PDGFRα promoter-driven Cre recombinase deleted loxP sites in mesangial cells in anti-GBM nephritis. (a) Immunohistochemical study for PDGFRα in glomeruli of anti-GBM nephritis in control mice. Veh., vehicle; GBM, anti-GBM nephritis. (b) Fluorescent studies for ZsGreen (green) and PDGFRβ (red), PECAM-1 (red) or podocin (red). ZsGreen was activated after removal of loxP-flanked stop codon by *Pdgfra* promoter-driven Cre recombinase. Anti-GBM nephritis was induced at 8 weeks of age and the mice were analyzed at 12 weeks of age. (c-e) Double fluorescent studies for ZsGreen (green) and PDGFRβ (red) (c), ZsGreen (green) and Megalin (red) (d), and ZsGreen (green) and AQP2 (red) (e). White arrows indicate double positive cells. Bar represents 50 μm.

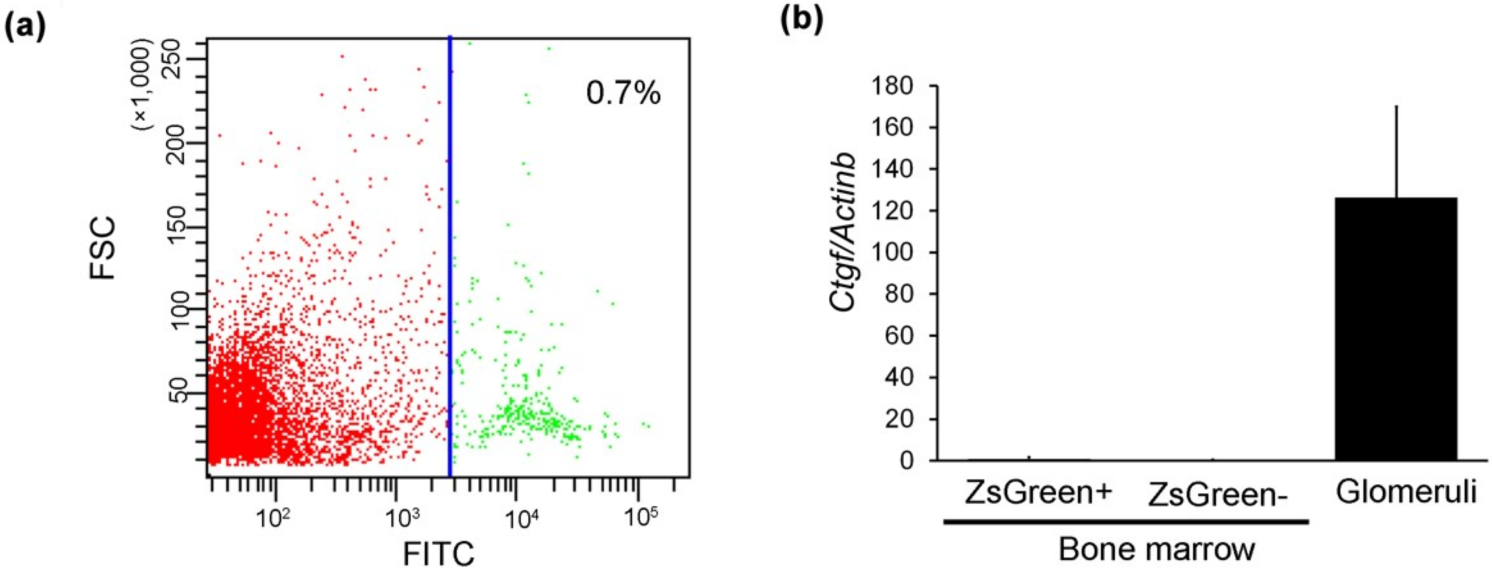

Supplementary Figure S5. Expression of *Ctgf* in cells isolated from a bone marrow of *Pdgfra*-ZsGreen mice. (a) Flow cytometric analysis of cells isolated from a bone marrow of *Pdgfra*-ZsGreen mice. FITC-positive or negative cells were sorted. (b) Real-time RT-PCR analysis for *Ctgf* expression in ZsGreen-positive or negative cells and glomeruli.

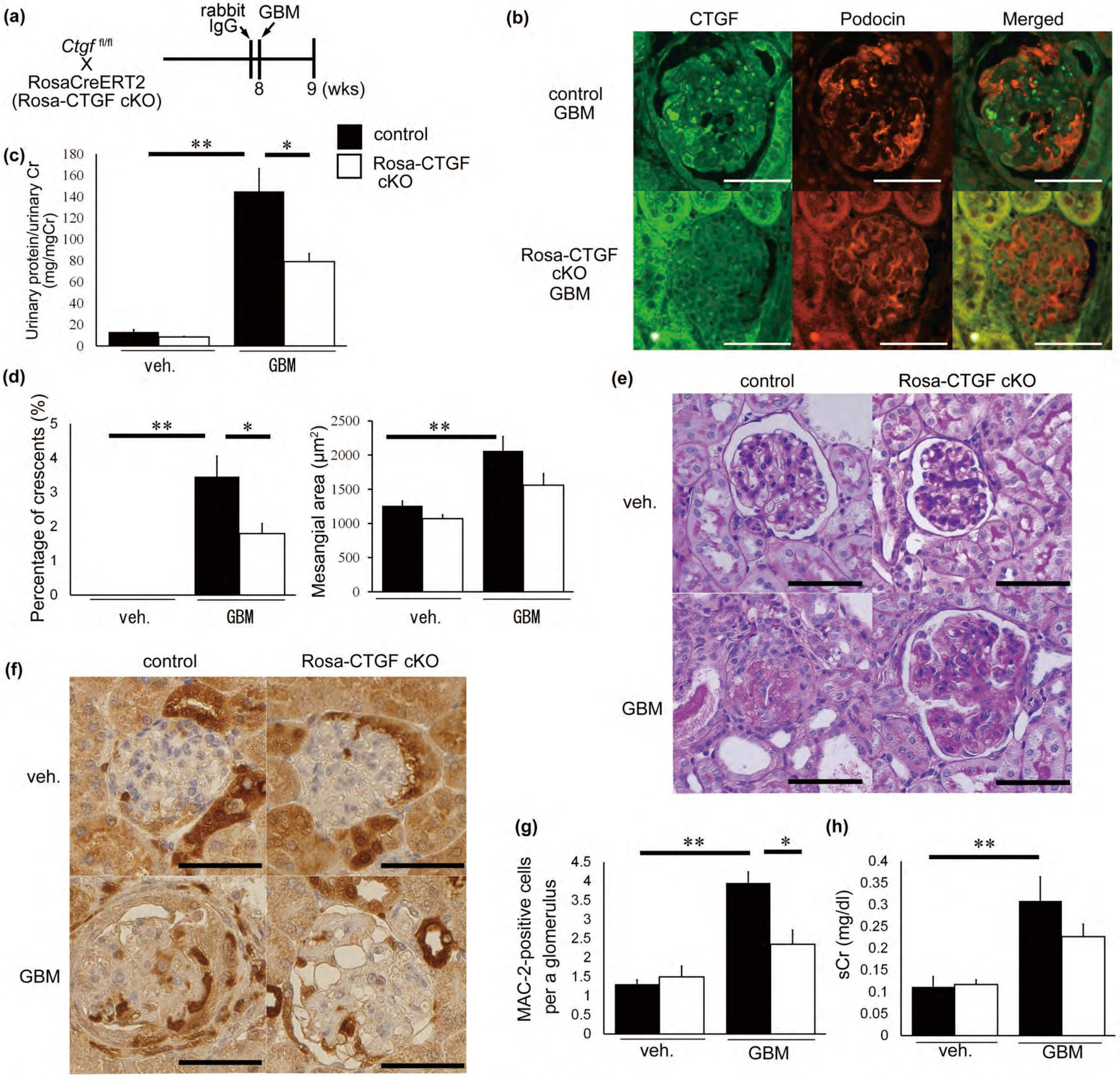

Supplementary Figure S6. Macrophage recruitment in Rosa-CTGF cKO mice with anti-GBM nephritis at the earlier stage. (a) An experimental protocol for the study on the anti-GBM nephritis in tamoxifen-inducible systemic CTGF cKO (Rosa-CTGF cKO) mice. Mice were treated with 4-OHT at 3 weeks of age, and then anti-GBM nephritis was induced at 8 weeks of age, and were analyzed at 1 week later. (b) Double immunofluorescence staining for CTGF (green) and podocin (red) in the glomeruli of control or Rosa-CTGF cKO mice at 1 week after induction of anti-GBM nephritis. Bar represents 50 μm. (c) Changes in proteinuria at 1 week after induction of anti-GBM nephritis. (d) The percentage of crescent formation (left panel) and the mesangial area (right panel) in anti-GBM nephritis at 1 week. (e) Representative photomicrographs of the kidneys at 1 week after induction of anti-GBM nephritis (PAS staining). Left upper panel, vehicle-treated control mice; right upper panel, vehicle-treated Rosa-CTGF cKO mice; left lower panel, control mice with anti-GBM nephritis; right lower panel show, Rosa-CTGF cKO mice with anti-GBM nephritis. Bar represents 50 μm. (f) Immunohistochemical studies for MAC-2 at 1 week after induction of anti-GBM nephritis. Bar represents 50 μm. (g) The number of MAC-2-positive cells at 1 week after induction of anti-GBM nephritis. (h) Serum creatinine level. Values are expressed as means ± s.e. $*P < 0.05$, $**P < 0.01$ vs. control GBM. Vehicle-treated control mice (n = 7), vehicle-treated Rosa-CTGF cKO mice (n = 7), control mice with GBM nephritis (n = 7), Rosa-CTGF mice with GBM nephritis (n = 9). Veh, vehicle; GBM, anti-GBM nephritis, closed boxes; control mice, open boxes; Rosa-CTGF cKO mice.

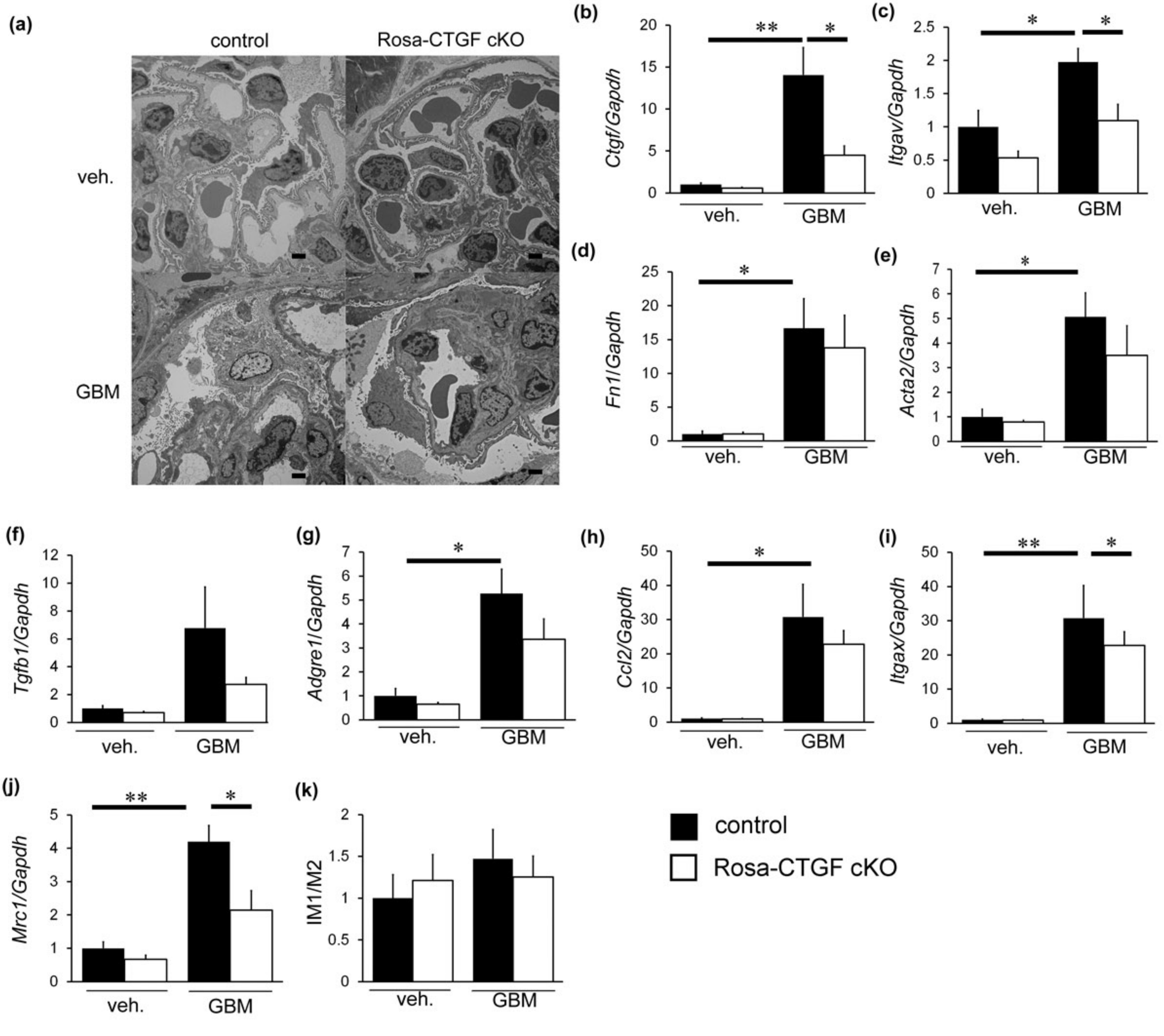

Supplementary Figure S7. Effects of CTGF deletion in anti-GBM nephritis at the earlier stage. (a) Electron microscopic analysis of a glomerulus in anti-GBM nephritis at 1 week after induction. Bar represents 2,000 nm. (b-j) Expression of *Ctgf* (b), *Itgav* (c), *Fn1* (d), *Acta2* (e), *Tgfb1* (f), *Adgre1* (g), *Ccl2* (h), *Itgax* (i) and *Mrc1* (j) mRNA in glomeruli. (k) The ratio of mRNA expression of M1/M2 macrophage markers in the glomeruli of Rosa-CTGF cKO mice. Values are expressed as means ± s.e. $^*P < 0.05$, $^{**}P < 0.01$ vs. control GBM. Vehicle-treated control mice (n = 7), vehicle-treated Rosa-CTGF cKO mice (n = 7), control mice with GBM nephritis (n = 7), Rosa-CTGF mice with GBM nephritis (n = 9). Veh, vehicle; GBM, anti-GBM nephritis, closed boxes; control mice, open boxes; Rosa-CTGF cKO mice.

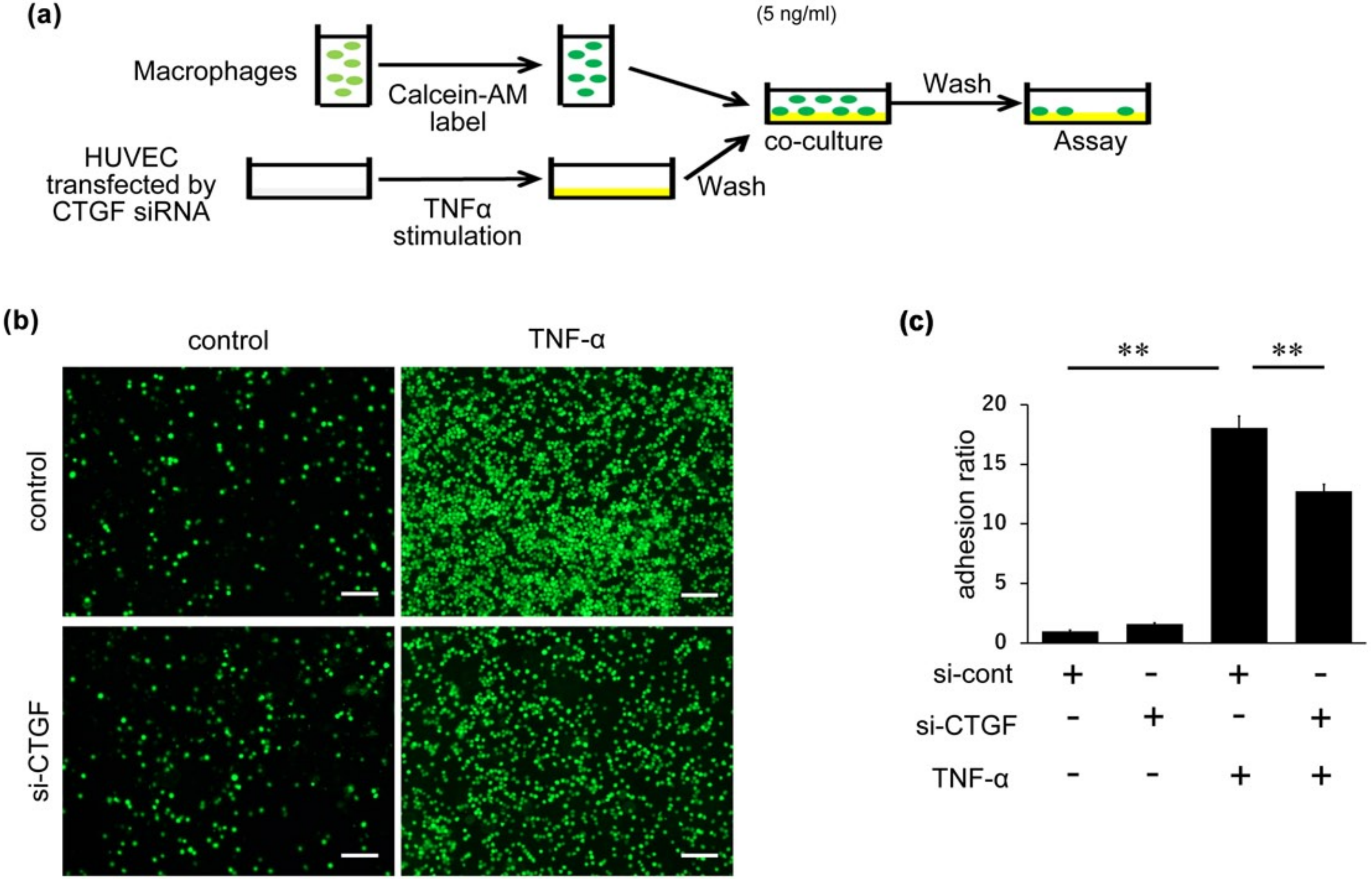

Supplementary Figure S8. CTGF mediates adhesion of macrophages to HUVEC on culture plates. (a) Experimental protocol of adhesion of fluorescein-dye-labeled RAW264.7 cells to 10 ng/ml TNF-α-stimulated HUVEC. (b) Inhibition of CTGF by siRNA reduced macrophage adhesion. Bar represents 100 μm. (c) Quantitative analyses of adhesion of RAW264.7 cells to HUVEC.

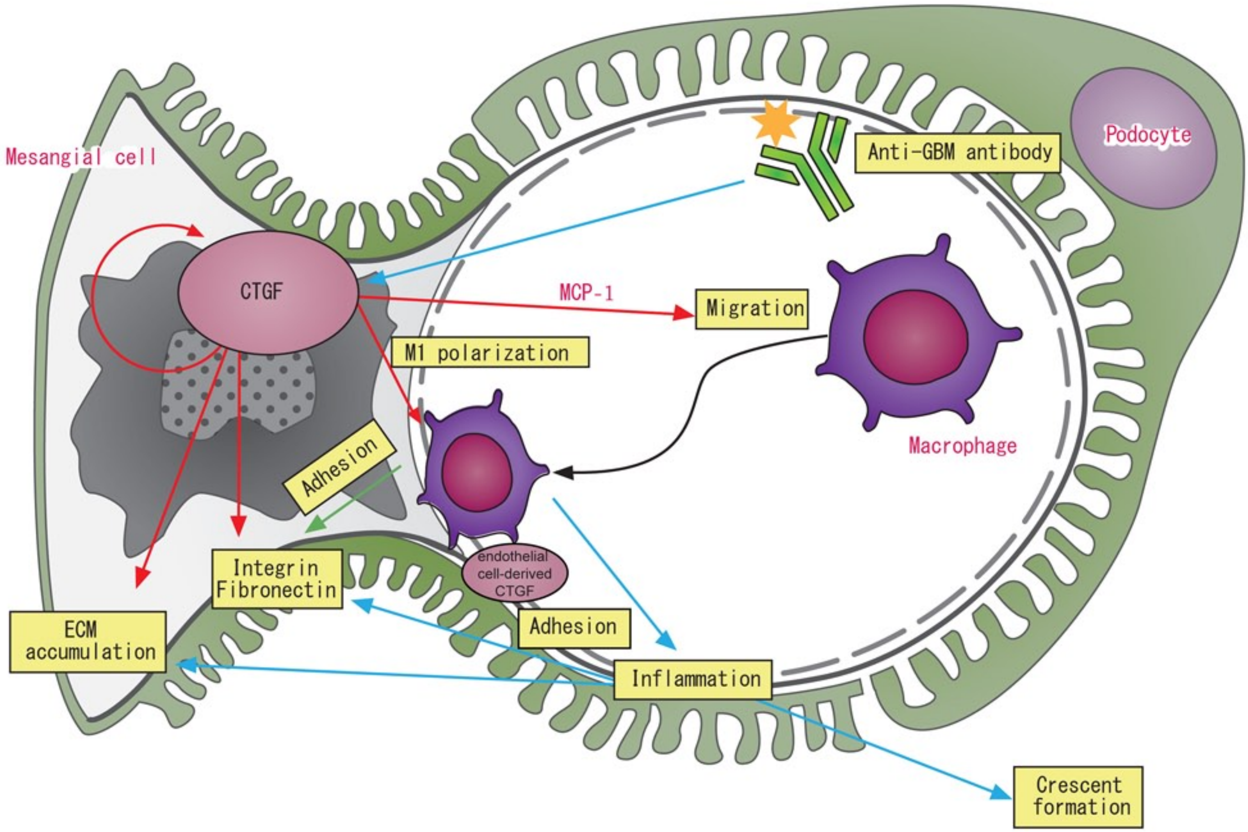

Supplementary Figure S9. CTGF mediates chemotaxis and adhesion of macrophages as well as ECM production in mesangial cells. Anti-GBM nephritis elicits upregulation of CTGF in mesangial cells. CTGF derived from endothelial cells is involved in macrophage adhesion. CTGF derived from mesangial cells increases MCP-1 (CCL2) expression, which induces macrophage migration and ECM proteins, including integrin αv and fibronectin, which contribute macrophage adhesion with mesangial cells.

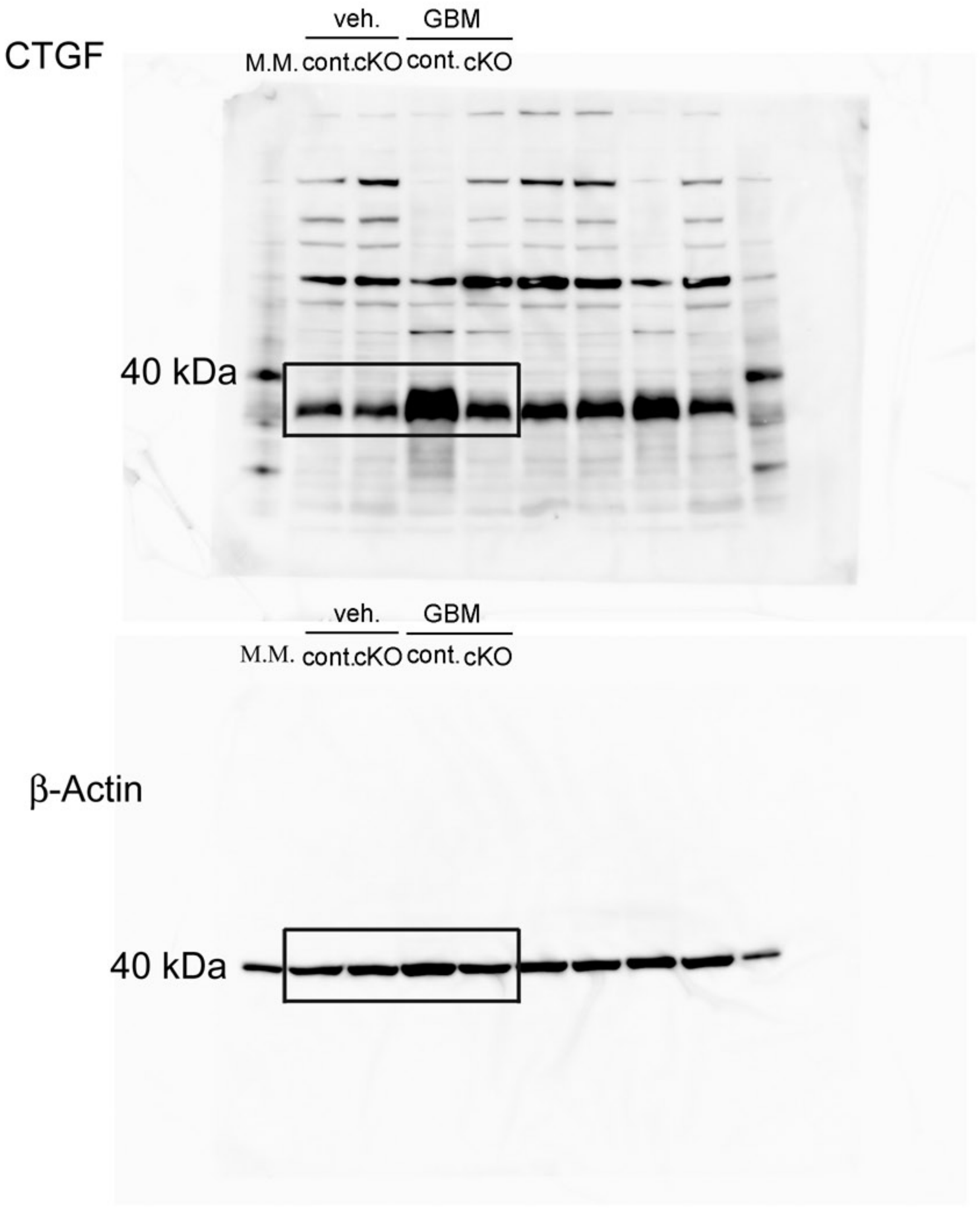

Supplementary Figure S10. Full-lenght blots in Figure 2d. Western blotting for CTGF and β-actin. M.M., molecular marker. Veh, vehicle; GBM, anti-GBM nephritis; cont. control mice; cKO, Rosa-CTGF cKO mice.

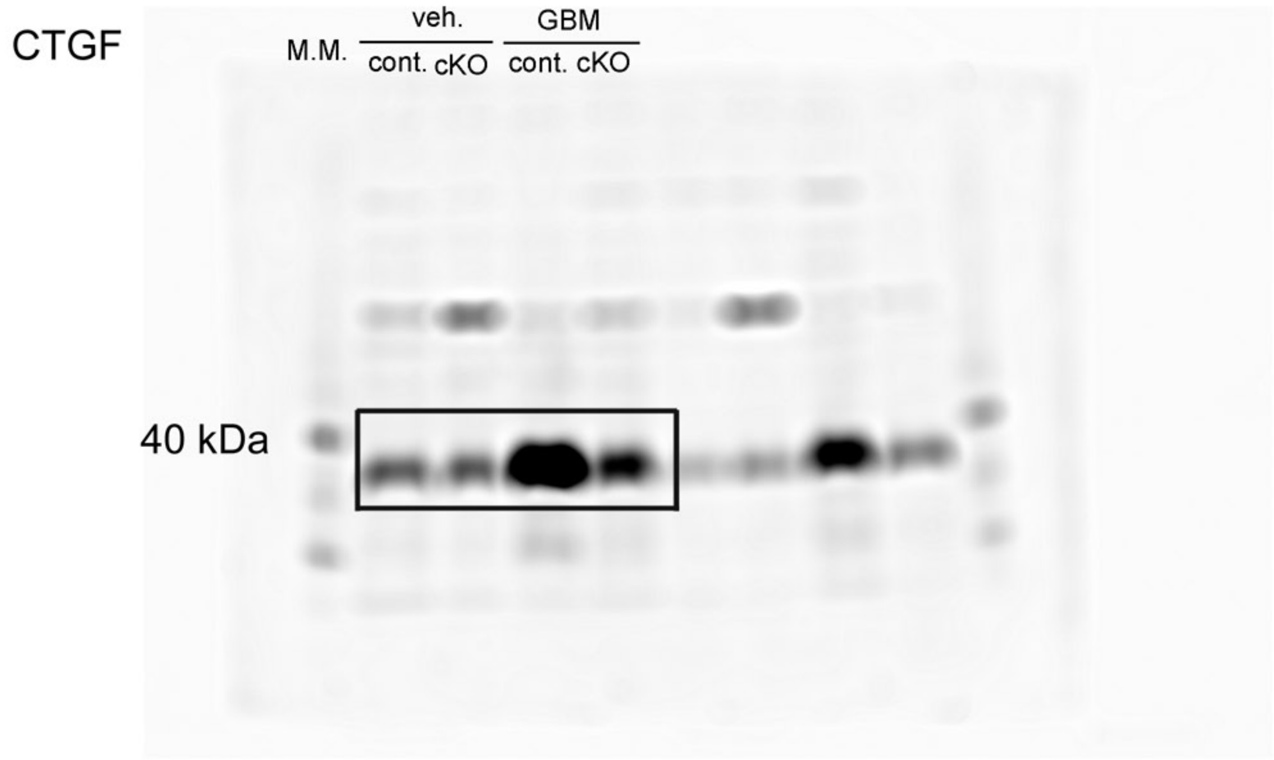

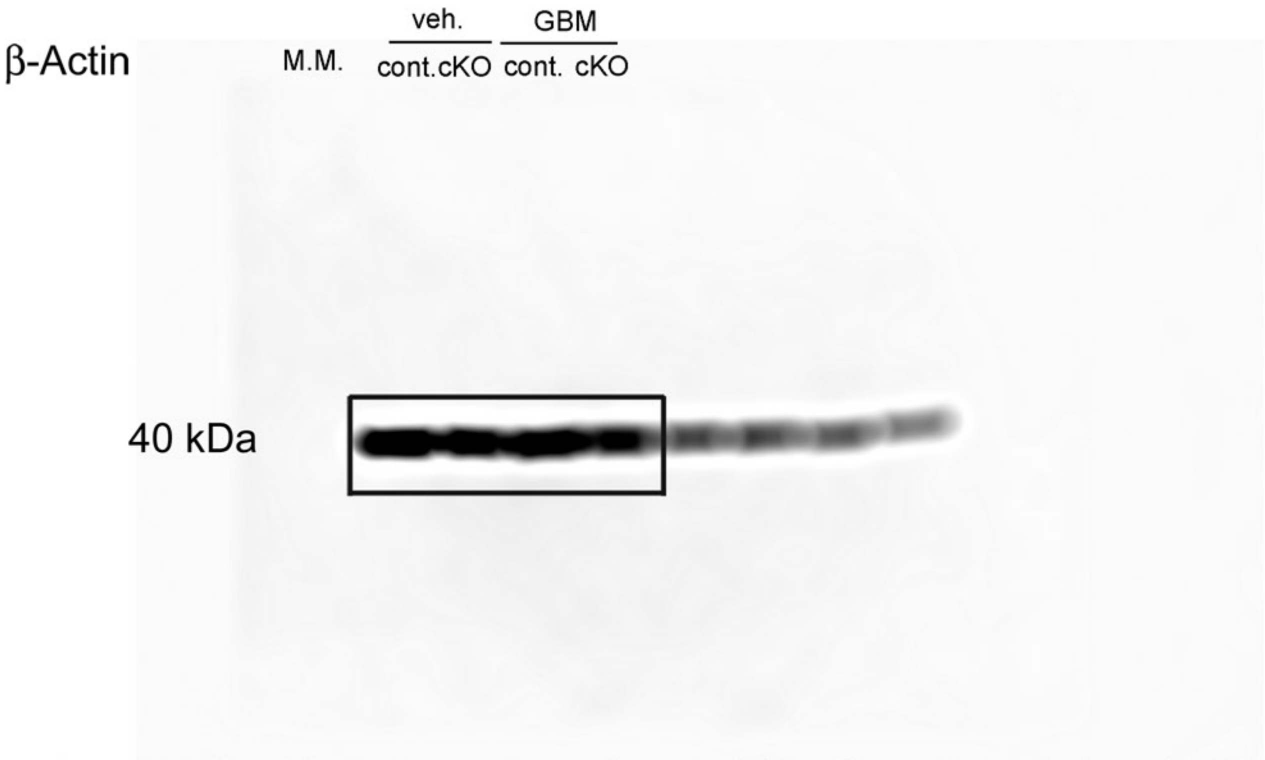

Supplementary Figure S11. Full-lenght blots in Figure 3j. Western blotting for CTGF and β-actin. M.M., molecular marker. Veh, vehicle; GBM, anti-GBM nephritis; cont. control mice; cKO, pod-CTGF cKO mice.

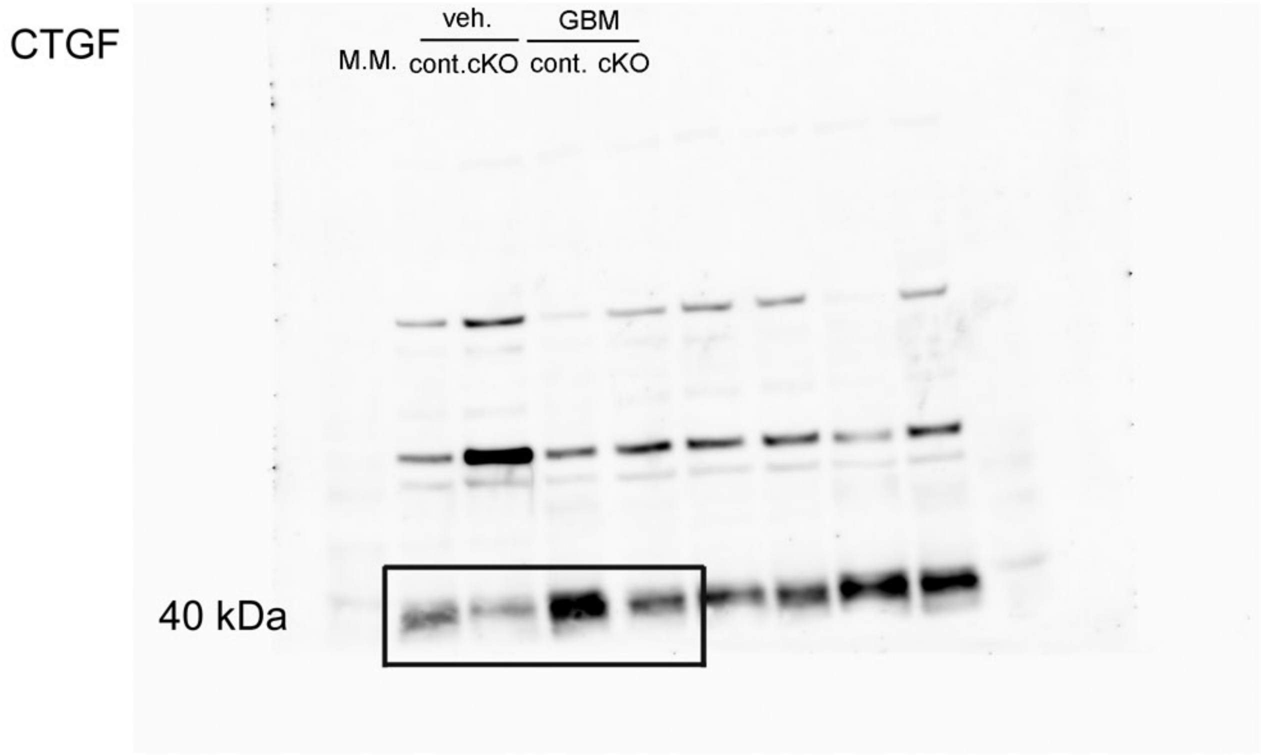

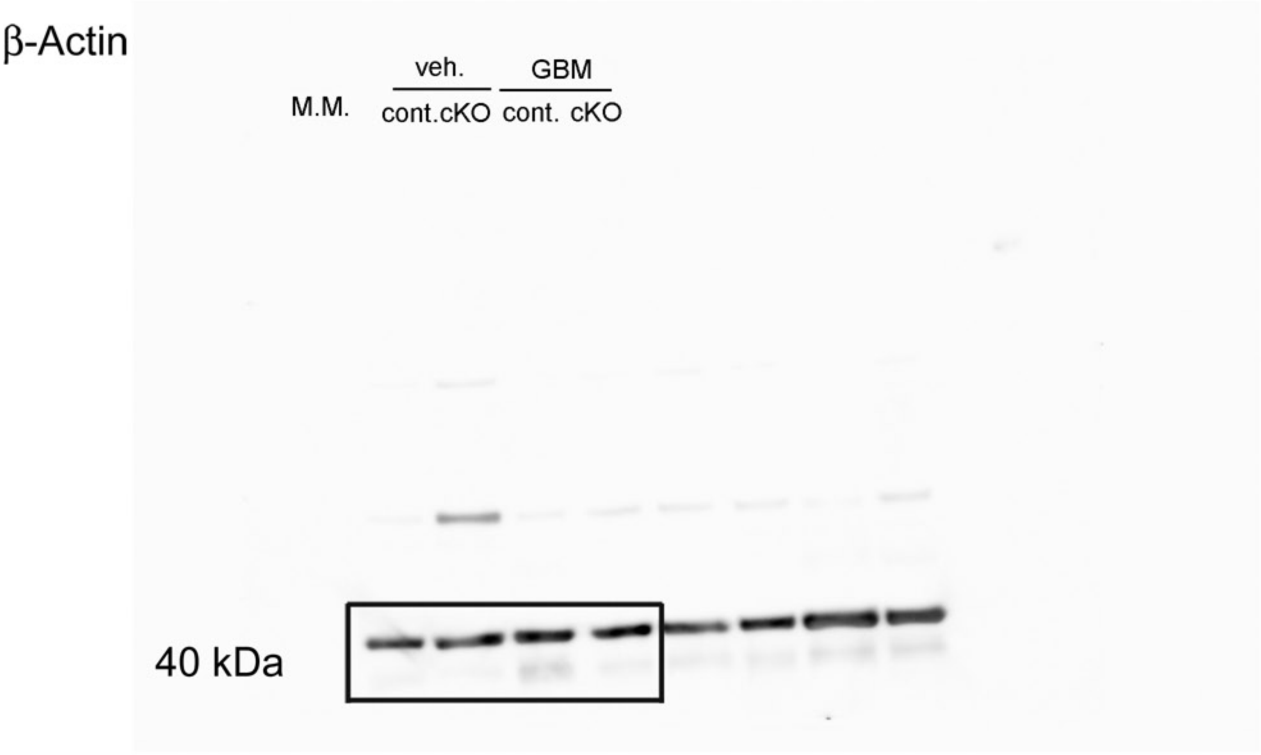

Supplementary Figure S12. Full-lenght blots in Figure 4j. Western blotting for CTGF and β-actin. M.M., molecular marker. Veh, vehicle; GBM, anti-GBM nephritis; cont. control mice; cKO, *Pdgfra*-CTGF cKO mice.

CTGF

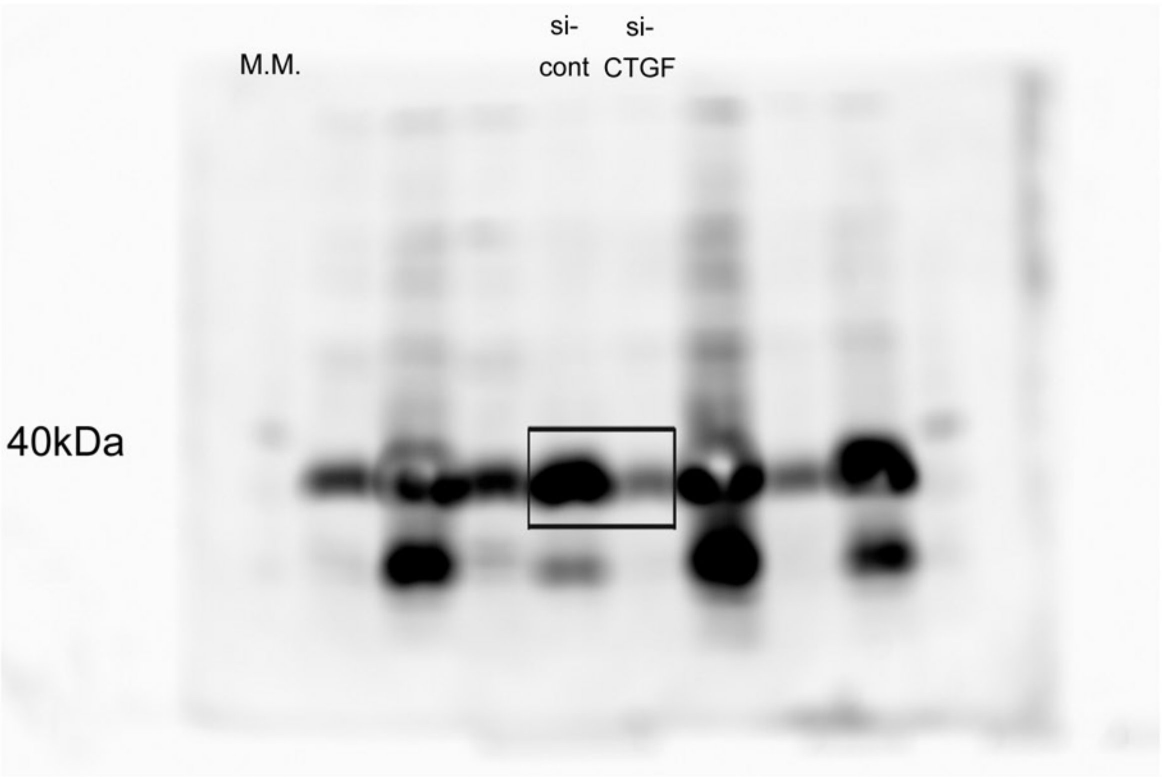

β-Actin

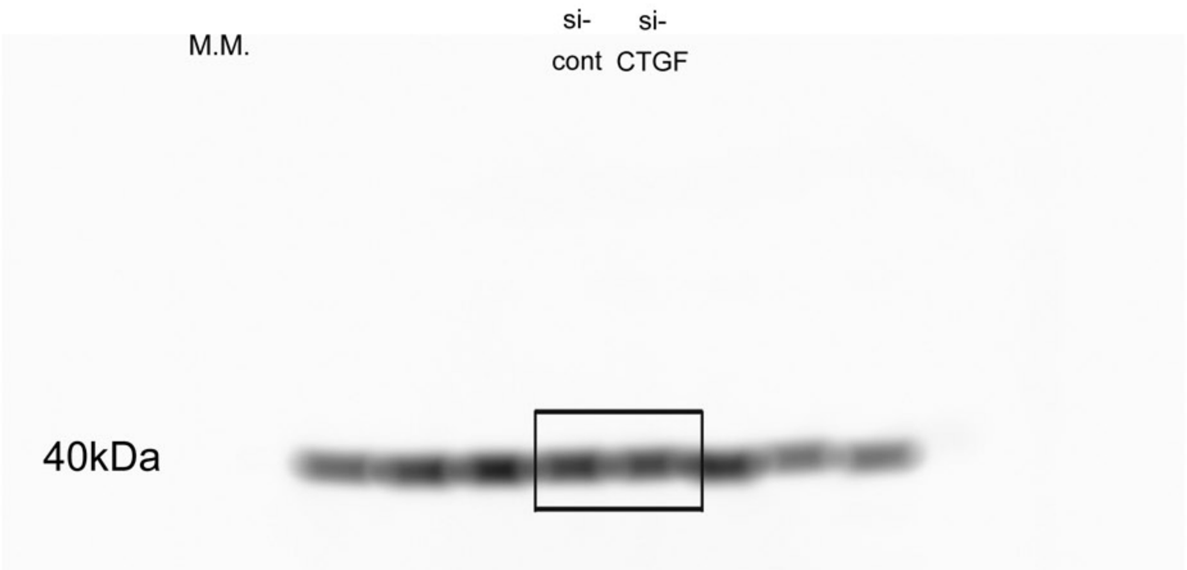

Supplementary Figure S13. Full-lenght blots in Figure 8d. Western blotting for CTGF and β-actin in Mes 13 cells. M.M., molecular marker. si-cont., siRNA for control; si-CTGF, siRNA against CTGF.

Supplementary Table S1. TaqMan primers and probe sequences

Gene	Forward primer	Reverse primer	Probe
Ctgf	5’-ttcccgagaagggtcaagct-3’	5’-tccttgggctcgtcacaca-3’	5’-FAM-cctgggaaatgctgcaaggagtgg-TAMRA-3’
Tgfb1	5’-gacgtcactggagttgtacgg-3’	5’-gctgaatcgaaagccctgt-3’	5’-FAM-agtggctgaaccaaggagacggaa-TAMRA-3’
Acta2	5’-cctgacgctgaagtatccgatag-3’	5’-ggtgccagatcttttccatgtc-3’	5’-FAM-acacggcatcatcaccaactggga-TAMRA-3’
Fn1	5’-atcatttcatgccaaccagtt-3’	5’-tcgcactggtagaagttcca-3’	5’-FAM-ccgacgaagagcccttacagttcca-TAMRA-3’
Itgav	5’-cagagagggagatgttcacactttg-3’	5’-gctcttgcctctatccagtcga-3’	5’-FAM-tggaatcgccaagtgcttgcagatcacc-TAMRA-3’
Adgre1	5’-tggtggtcataatctctgcttctg-3’	5’-agacaggccccaggaaactc-3’	5’-FAM-cccgtctctgtattcaaccagcagcgatt-TAMRA-3’
Ccl2	5’-cggctggagcatccacgt-3’	5’-attgggatcatcttgctggtgaat-3’	5’-FAM-tcagccagatgcagttaacgccccac-TAMRA-3’
Itgax	5’-cctggatagcctttcttc-3’	5’-cgtccatgtgaaaatgtg-3’	5'-FAM-ttcttgtcttggcttcaacttgga-TAMRA-3'
Mrc1	5’-aggagttcattatacaaactg-3’	5’-caccaatcacaactacac-3’	5’-FAM-taggctacttcttcttccaccagg-TAMRA-3’
Icam1	5’- ttgagaactgtggcaccgtg-3’	5’-tctcagctccacactctccg-3’	5’-FAM-tcgtccgcttccgctaccatcaccg-TAMRA-3’
Vcam1	5’-cgctaggttacacagtggtgaa -3’	5’-ctgacccagatggtggtttcc-3’	5’-FAM-aacccaaacagaggcagagtgtacagcc -TAMRA-3’

Ctgf, connective tissue growth factor; *Tgfb1*, transforming growth factor-beta 1; *Fn1*, fibronectin; *Acta2*, alpha smooth muscle actin; *Itgav*, integrin alpha V; *Adgre1*, adhesion G protein-coupled receptor E1; *Ccl2*, chemokine ligand 2; *Itgax*, integrin alpha X; *Mrc1*, mannose receptor C type 1; *Icam1*, intercellular adhesion molecule 1; *Vcam1*, vascular cell adhesion molecule 1.
